# Characterization of a *Nicotiana tabacum* phytochelatin synthase 1 and its response to cadmium stress

**DOI:** 10.3389/fpls.2024.1418762

**Published:** 2024-08-29

**Authors:** Chanjuan Wu, Jie Zhang, Mei Chen, Jikai Liu, Yunlai Tang

**Affiliations:** School of Life Science and Engineering, Southwest University of Science and Technology, Mianyang, China

**Keywords:** phytochelatin synthase, gene expression, phytochelatins, glutathione, cadmium tolerance

## Abstract

Phytochelatin synthase (PCS) is a critical enzyme involved in heavy metal detoxification in organisms. In this study, we aim to comprehensively investigate the molecular and functional characteristics of the *PCS1* gene from *Nicotiana tabacum* by examining its enzymatic activity, tissue-specific expression pattern, Cd-induced expression, as well as the impact on Cd tolerance and accumulation. The results demonstrated that the amino acid sequence of NtPCS1 shared a high similarity in its N-terminal region with PCS from other species. The enzymatic activity of NtPCS1 was found to be enhanced in the order Ag^2+^ > Cd^2+^ > Cu^2+^ > Pb^2+^ > Hg^2+^ > Fe^2+^ > Zn^2+^. In addition, RT-PCR data indicated that *NtPCS1* gene is constitutively expressed, with the highest expression observed in flowers, and that its transcript levels are up-regulated by CdCl_2_. When tobacco overexpressing *NtPCS1* (PCS1 lines) were grown under CdCl_2_ stress, they produced more phytochelatins (PCs) than WT plants, but this did not result in increased Cd accumulation. However, in a root growth assay, the PCS1 lines exhibited hypersensitivity to Cd. The overexpression of *NtPCS1* itself does not appear to be the primary cause of this heightened sensitivity to Cd, as the *Arabidopsis thaliana Atpcs1* mutant overexpressing *NtPCS1* actually exhibited enhanced tolerance to Cd. Furthermore, the addition of exogenous glutathione (GSH) progressively reduced the Cd hypersensitivity of the PCS1 lines, with the hypersensitivity even being completely eliminated. Surprisingly, the application of exogenous GSH led to a remarkably enhanced Cd accumulation in the PCS1 lines. This study enriches our understanding of the molecular function of the *NtPCS1* gene and suggests a promising avenue for Cd tolerance through the heterologous expression of *PCS* genes in different species.

## Introduction

Plants can produce thiol peptides, such as metallothioneins (MTs), glutathione (GSH), and phytochelatins (PCs), to detoxify or maintain metal ion homeostasis (Rea, 2012; Seregin and Kozhevnikova, 2023). PCs are a class of small peptides that bind heavy metals, characterized by the general structure (γ - Glu - Cys)_n_ - Gly, where n varies from 2 to 11 (Sharma et al., 2016; Seregin and Kozhevnikova, 2023). They are enzymatically synthesized from GSH by phytochelatin synthase (PCS) (Rea, 2012; Seregin and Kozhevnikova, 2023). These peptides chelate heavy metal ions, forming stable complexes in the cytosol, which are subsequently transported into the vacuole (Rea, 2012; Sharma et al., 2016; Seregin and Kozhevnikova, 2023).

Genes encoding PCS have been identified in various species, such as *Schizosaccharomyces pombe* (Ha et al., 1999; Shine et al., 2015), *Caenorhabditis elegans* (Kuhnlenz et al., 2015), *Ancylostoma ceylanicum* (Rigouin et al., 2013), *Ceratophyllum demersum* (Shri et al., 2014), *Marchantia polymorpha* (Degola et al., 2014), *Pteris vittate* (Dong et al., 2005), *A. thaliana* (Vatamaniuk et al., 1999; Kuhnlenz et al., 2014), *Triticum aestivum* (Wang et al., 2012), *Oryza sativa* (Park et al., 2019), and *Brassica juncea* (Liu et al., 2021). *PCS* genes from different plant species respond differently to metal treatments. A study on the effects of Cu, Zn, Ni, and Cd on *Azolla* species revealed that *PCS1* gene expression was induced by these heavy metals, with *A. pinnata* showing the highest Cu and Cd uptake, while *A. filiculoides* and samples from the Anzali wetland showed the highest Ni and Zn uptake, indicating species- and metal-specific expression of the *PCS* gene (Talebi et al., 2019). In addition, the transcript level of *BjPCS* was upregulated in *B. juncea* in response to Cd and As (Heiss et al., 2003; Ahmad and Gupta, 2013). In *O. sativa*, *OsPCS7* expression was induced by Hg and Pb, whereas *OsPCS9* was stimulated by Cd and Zn; furthermore, both *OsPCS5* and *OsPCS15* were activated by Cd and As (Park et al., 2019). In *Morus notabilis*, the expression of *MnPCS1* and *MnPCS2* was significantly enhanced, with Cd showing a stronger effect than Zn (Fan et al., 2018). In *Solanum lycopersicum*, *SlPCS1* was more strongly induced by Cd and Pb than by Cu (Kısa, 2019). In *Saccharum officinarum*, the expression of *SoPCS* was increased in Cd-treated roots, whereas the expression pattern in leaves was irregular (Yousefi et al., 2018). However, the transcript levels of *A. thaliana AtPCS1/2*, *Thlaspi caerulescens TcPCS1/2*, and *Brassica rapa BrPCS1/2* did not differ significantly between the control and Cd-treated conditions, suggesting that their expression is not influenced by Cd treatment (Ha et al., 1999; Lee et al., 2002; Meyer et al., 2011; Liu et al., 2021). Moreover, *PCS* gene expression can also be affected by other factors. For example, in *T. aestivum*, the expression of *TaPCS* was upregulated in response to infections by *Pseudomonas gessardii* and *Brevundimonas intermedia* (Soto et al., 2019). The supplementation of Na_2_SO_4_ as an additional sulfur source in the growth medium resulted in an elevation of *OsPCS* gene expression and PCs content in *O. sativa* (Cao et al., 2018).

In plants, PCs play a crucial role in metal tolerance, particularly against Cd. It is anticipated that enhancing the expression of *PCS* genes or introducing them heterologously could improve the tolerance and accumulation of heavy metals. This effect has been observed not only in various plant species but also in yeast and *Escherichia coli*. Overexpression of the *A. thaliana AtPCS1* gene in *E. coli* and *Saccharomyces cerevisiae* has been demonstrated to increase Cd tolerance and accumulation (Vatamaniuk et al., 1999; Sauge-Merle et al., 2003). Overexpression of mulberry *MnPCS1*/*2* in *A. thaliana* and tobacco has resulted in increased tolerance to Zn and Cd tolerance (Fan et al., 2018). The *BnPCS1* gene from *Boehmeria nivea* has been found to enhance Cd tolerance, accumulation, and translocation in *A. thaliana* when overexpressed (Zhu et al., 2021). Overexpression of three duplicated *BnPCS* genes from *Brassica napus* and the *C. elegans CePCS* gene rescued the deficiency in phytochelatin synthesis and Cd sensitivity of the *AtPCS1*-deficient *cad1-3* mutant, leading to enhanced accumulation and translocation in *A. thaliana* (Kuhnlenz et al., 2014, 2015; Bai et al., 2019; Inoue et al., 2022). Furthermore, the expression of *AtPCS1* in *B. juncea* enhanced tolerance to Cd, Zn, and As (Gasic and Korban, 2007a; 2007b). *Nicotiana glauca* expressing wheat *TaPCS1* exhibited enhanced tolerance and accumulation to Cd and Pb (Martinez et al., 2006). Yeast strains expressing the *B. rapa BrPCS1* gene have demonstrated increased tolerance to Cd and Zn (Liu et al., 2021). However, some studies have revealed that *PCS* transgenic plants exhibit reduced tolerance to Cd and Zn. For example, overexpression of *AtPCS1* in *A. thaliana* and tobacco led to Cd hypersensitivity despite increased PCs production (Lee et al., 2003a, 2003; Li et al., 2004; Wojas et al., 2008). The heterologous expression of wheat *TaPCS1* in rice increased Cd sensitivity (Wang et al., 2012). Similar results were observed in transgenic *A. thaliana* plants that overexpressed the rice *OsPCS5* and *OsPCS15* genes (Park et al., 2019). Thus, the effects of overexpressing or heterologously expressing *PCS* genes on metal tolerance may vary between species, highlighting the need for species-specific studies to understand PCS-mediated metal homeostasis in plants.

In addition to their well-established roles in metal stress response, *PCS* genes participate in a variety of biological processes. The *AtPCS1*-deficient *A. thaliana cad1-3* mutant exhibited a marked cell death phenotype in response to infections by the pathogen *Phytophthora infestans* and *Pseudomonas syringae*, respectively, suggesting that *AtPCS1* is essential for defense against bacterial pathogens (Kuhnlenz et al., 2015; De Benedictis et al., 2018). *AtPCS1* is also involved in the regulation of callose deposition and auxin content (Clay et al., 2009; De Benedictis et al., 2018; Hématy et al., 2020). *AtPCS2* is known to plays a role in the response to salt stress (Kim et al., 2019). Furthermore, PCS function as a cysteine peptidase to regulate the catabolism of glutathione and glutathione conjugates in the cytosol (Blum et al., 2007, 2010; Inoue et al., 2022). Moreover, *PCS* is involved in the maintenance of Fe homeostasis in *Nitella mucronata* (Fontanini et al., 2018).

In general, GSH and PCs are involved in the mechanisms of metal detoxification and transport, but are not implicated in the mechanisms of metal hyperaccumulation (Sharma et al., 2016). A previous study showed that the expression levels of *PCS* genes are higher in *A. thaliana* (*AtPCS1/2*) compared to those in the hyperaccumulator species *Arabidopsis helleri* (*AhPCS1/2*) and *T. caerulescens* (*TcPCS1/2*) (Meyer et al., 2011). The PCs content in the shoots of hyperaccumulators such as *A. halleri*, *Sedum alfredii*, and *Noccaea caerulescens* is low or even completely absent (Wójcik et al., 2006; Sun et al., 2007; Meyer et al., 2011). *Dianthus carthusianorum* plants grown in non-metalliferous soil have a higher level of PCs compared to those grown in metalliferous soil (Wójcik et al., 2015), and a similar phenomenon is observed in other species such as *S. alfredii*, *Silene vulgaris*, and *Dettrichia viscose* (De Knecht et al., 1994; Fernández et al., 2014; Wójcik et al., 2015). This suggests that the high tolerance of hyperaccumulators to metals is not due to an increase in PC biosynthesis. Since hyperaccumulators have highly efficient PC-independent metal detoxification pathways in their shoots, *PCS* is not activated there, which may be advantageous given the high energetic cost of PC biosynthesis (Ebbs et al., 2002; Meyer et al., 2011). In contrast, in non-accumulators like *Arabidopsis lyrata*, Cd primarily binds to sulfur-containing ligands, indicating that GSH and PCs are involved in Cd detoxification in non-accumulator plants (Isaure et al., 2015). The more efficient PC biosynthesis in non-accumulators may be associated with higher expression levels of the *PCS* gene, as well as a higher availability of metal ions in the roots of these species compared to hyperaccumulators (Meyer et al., 2011).

To date, several studies have reported on the tobacco *NtPCS* gene. These studies indicate that overexpression of the *NtPCS1* gene in *Saccharomyces cerevisiae* enhanced tolerance and accumulation of Cd and improved resistance to As (Kim et al., 2005). Tobacco plants that expressed *NtPCS1* demonstrated increased tolerance to both Cd and As without altering the accumulation of these metals. Moreover, tobacco plants expressing *antisense-NtPCS1* exhibited growth retardation during the early stages, suggesting a role in plant development (Lee and Hwang, 2015). In this study, to comprehensively investigate the molecular characteristics of the *NtPCS1* gene and its functions in metal detoxification across various species, we examined the tissue-specific expression pattern, Cd-induced expression, enzymatic activity, Cd tolerance and accumulation in *E. coli*, *A. thaliana*, and tobacco plants expressing *NtPCS1*, as well as the influence of GSH on Cd accumulation in tobacco plants.

## Materials and methods

### Plant materials and growth conditions

All *N. tabacum* seeds used in this study are in NC89 ecotype. The wild-type (WT) and homozygous transgenic tobacco were grown in the greenhouse at 25°C for 16 h in the light and 8 h in the dark, at a light intensity of 250 μmol·m^-2^·s^-1^. The suitable humidity is 70%.

All *A. thaliana* seeds used in this study are in Col-0 ecotype. The Col-0, *Atpcs1* mutant (SAIL-650-C12) and homozygous transgenic *A. thaliana* plants were grown in the greenhouse at 22°C for 16 h in the light and 8 h in the dark, at a light intensity of 100 μmol·m^-2^·s^-1^. The suitable humidity is 60%.

### Plant expression constructs and transgenic plants

Genomic DNA was extracted from 30-day-old tobacco seedlings by CTAB (C8440-25I, SOLARBIO) method. To isolate *NtPCS1* promoter from genomic DNA, PCR primers were designed. The forward primer 5’-AAGCTTGTGCAGCAGCTGTTGAAGAAAG-3’ with a *Hin*d III site and the reverse primer 5’-GGATCCTTTTTCTCGCTTCAGAATCTCC-3’ with a *Bam*H I site were used. The location of forward primer was 1,097 bp upstream of the translation start site. The *NtPCS1* promoter was amplified by PCR and ligated to pBI121 vector, replacing the cauliflower mosaic virus CaMV35S promoter. The resulting constructs consisted of the *GUS* gene driven by the *NtPCS1* promoter (pBI121-*NtPCS1pro::GUS*).

Total RNA from 30-day-old tobacco seedlings was extracted using RNAprep Pure Plant Kit (DP432, TIANGEN), and cDNA was obtained by reverse transcriptase reaction using 5×All-In-One kit (G492, Abmart). One microliter of cDNA was used for a PCR reaction. The forward primer 5’- GGATCCATGGCGATGGCGGGTTTA -3’ with a *Bam*H I site and the reverse primer 5’- GTCGACCTAGAAGGGAGGTGCAGCTAAA -3’ with a *Sal* I site were used. The 1,506 bp fragment obtained by PCR was ligated to pBI121 vector under the transcriptional control of the CaMV35S promoter. The resulting constructs consisted of the *NtPCS1* gene driven by the CaMV35S promoter (pBI121-*35Spro::NtPCS1*).

Both pBI121-*NtPCS1pro::GUS* and pBI121-*35Spro::NtPCS1* were transferred separately to *Agrobacterium tumefaciens* strain *EHA105* and the recombinant strains were used to transfect tobacco by standard leaf disc transformation method to obtain corresponding transgenic tobacco.

### *NtPCS1* expression pattern analysis

To assess the expression pattern of the *NtPCS1* gene, seeds from both WT and homozygous transgenic tobacco, which had been transformed with the pBI121-*NtPCS1pro::GUS* construct, were germinated and grown on 1/2 MS medium for one month. Subsequently, the seedlings were transferred to soil for continued growth, allowing them to progress to the flowering and fruiting stage. From WT, all root, stem, and leaf tissues from 40-day-old plants, all flowers from 3-month-old plants during the flowering period, and all seeds from 5-month-old mature plants were collected for RT-PCR to detect the transcript level of *NtPCS1*. In addition, 10-day-old and 1-month-old seedlings, as well as tissues from 3-month-old transgenic plants including root, stem, leaf, stigma, anther, ovary, petal, and sepal, were harvested for GUS histochemical staining.

### Quantitative RT-PCR analysis

Total RNA from WT and homozygous transgenic tobacco was extracted using RNAprep Pure Plant Kit (DP432, TIANGEN), and cDNA from 2 μg of total RNA was obtained by reverse transcriptase reaction using 5×All-In-One kit (G492, Abmart). We then performed RT-PCR using the KAPA SYBR FAST qPCR Master kit (KAPA, KK4601). To analyze transcript level of *NtPCS1* in WT and transgenic tobacco, the specific primers (5’TGGTCTTGAATGCCCTTGC 3’ and 5’GAGGCTCACAACAGTCCAACA 3’) were designed for RT-PCR. The RT-PCR product was gained after 40 cycles. The *NtRL2* gene (Ribosomic protein L2, GenBank Z14081, 5’GTAAGGGAGCGGGTTCAGTCT 3’ and 5’AACGGAGCACCCCTACCTG 3’) was used as a control (Liu et al., 2019).

### GUS enzymatic activity

To analyze the activity of the *NtPCS1* promoter induced by Cd treatment, homozygous transgenic tobacco seeds transformed with the pBI121-*NtPCS1pro::GUS* construct were germinated on 1/2 MS medium supplemented with 0 or 100 µM CdCl_2_. After 10 and 20 days, seedlings were harvested for GUS enzymatic activity and histochemical staining.

Collected samples were homogenized in liquid nitrogen and suspended in 200 µL GUS extraction buffer (0.1 M Phosphate buffer pH 7.0, 10% (w/v) SDS, 0.5 M EDTA, pH 8.0, Triton X-100, β-mercaptoethanol), and the soluble protein fraction was collected after centrifugation at 12,000 rpm. Quantitative fluorometric analysis of GUS enzymatic activity was carried by a luminescence spectrometer (LS-55, PerkinElmer) after 60 min incubation with the GUS substrate. The protein content was determined using the Bradford protein assay. Specifically, a series of bovine serum albumin (BSA) solutions were prepared with concentrations of 0, 5, 10, 20, 40, and 80 μg/ml. The absorbance of these solutions at 595 nm was measured using a UV spectrophotometer (UV-8000, METASH), and a standard curve was constructed to illustrate the correlation between protein concentration and absorbance values. Subsequently, the absorbance of the samples incubated with G-250 solution was determined at 595 nm using a UV spectrophotometer (UV-8000, METASH), and the protein concentration was calculated using the established standard curve.

### GUS histochemical staining

For GUS staining, samples were incubated in a staining solution containing 50 mM NaH_2_PO_4_ at pH 7.0, 50 mM Na_2_HPO_4_ at pH 7.0, 0.5 mM K_3_[Fe(CN)_6_], 0.5 mM K_4_[Fe(CN)_6_], 10 mM EDTA at pH 8.0, 0.1% (v/v) Triton X-100, 20% (v/v) methanol, and 1 mM 5-bromo-4-chloro-3-indolyl glucuronide (X-gluc). This incubation was performed overnight at 37°C. After the incubation, samples were then subjected to a series of ethanol solutions with gradually increasing concentrations (50%, 70%, 80% and 90%) for chlorophyll decolorization. The samples were incubated for 30 minutes at 37°C under each ethanol concentration, and subsequently, photographs were taken using a stereomicroscope (OLS5100, LEXT).

### *PCS1* expression in *E. coli*

The full coding sequence (CDS) of the *NtPCS1* gene amplified by PCR was ligated into pET28a and transformed into *E. coli* strain *BL21(DE3)*. The transformed bacteria were cultured at 37°C until the OD_600_ reached 0.6. Subsequently, 200 µM isopropyl β-D-1-thiogalactopyranoside (IPTG) was added, and the bacteria were grown at 15°C for 12 hours to induce protein expression. An extract was obtained by sonicating the collected bacteria in buffer (50 mM Tris-HCl pH 7.9, 14% [v/v] glycerol, and 10 mM β- mercaptoethanol), and the recombinant NtPCS1 protein was purified by a Ni-affinity column. The purified protein was used for PCS enzyme activity assay.

In addition, the *NtPCS1* cDNA was ligated to pTrc99A, and transformed into *E. coli* strain *DH5α*. The transformed bacteria were cultured at 37°C until the OD_600_ reached 0.6. Subsequently, 100 µM IPTG and various CdCl_2_ concentrations (0, 50,100, 500, 1000 µM) were added, and the bacteria were grown at 37°C for an additional 3 hours. The OD_600_ values and intracellular Cd content were measured by UV spectrophotometer (UV-8000, METASH) and AAS (AA 700, PerkinElmer) analysis, respectively.

### Phytochelatin synthase enzyme assay

PCS enzyme activity was performed by a modified protocol according to a method previously described (Chen et al., 1997). The assay mixture contained 30 µg recombinant NtPCS1 protein, 200 mM Tris-HCl pH 8.0, 10 mM β- mercaptoethanol, 10 mM GSH, and 100 µM CdCl_2/_ZnSO_4/_CuSO_4/_AgNO_3/_FeSO_4/_HgCl_2/_MnCl_2/_MgSO_4/_Pb(NO_3_)_2/_CoCl_2_, respectively, in a total volume of 300 µl. In detail, the mixture without metal ions was incubated at 35°C for 5 min, and the action was started by adding metal ions. After 15 min, the action was stopped by adding 30 ul 50% 5-sulfosalicylic acid. Then the solution was incubated on ice for 10 min. The protein left over was removed by centrifugation at 13,000 rpm for 10 min, and the supernatant was used for PCs assay. PCs were analyzed by HPLC (1200LC/MS, Varian) as previously described (Sneller et al., 2000). The PCS activity is quantified as the total number of nmols of γ - Glu - Cys transferred per minute per mg of the NtPCS1 protein as previously described (Chen et al., 1997). In this study, since the general structure of PCs is (γ - Glu - Cys)n - Gly, where n ranges from 2 to 4, the PCS activity was calculated as follows:


PCS activity = 1×(nmol of PC2) + 2×(nmol of PC3) + 3×(nmol of PC4).

### Tobacco seedling experiments

In this study, three similar seedling experiments were performed, each repeated three times independently, and are described as follows:

(i) To analyze the expression of *NtPCS1* induced by Cd treatment, seeds from WT were germinated on a foam sheet. After 28 days, the seedlings were transferred to holes in a plastic septum within blue boxes, allowing only the roots to be submerged in a 1/2 Hoagland solution. The Hoagland solutions were refreshed every 2 to 3 days. Subsequently, after 7 days, the tobacco seedlings were moved to a fresh solution containing varying concentrations of CdCl_2_ (0, 30, 60, 100, 200 µM). After 48 hours, shoot and root samples were collected for RT-PCR to detect the transcript level of *NtPCS1*.

(ii) Transgenic tobacco transformed with pBI121-*35Spro::NtPCS1* construct was named PCS1 lines. In this study, 12 transgenic lines were analyzed for the expression of *NtPCS1*, and three lines (PCS1-1, PCS1-8 and PCS1-11) that exhibited altered expression levels of *NtPCS1*, with the expression levels being higher than those in WT, were selected for further experiments. Seeds from both WT and homozygous PCS1 lines, which overexpressed *NtPCS1*, were germinated on 1/2 MS medium containing 0, 50, 100, 150, and 200 µM CdCl_2_, either in the absence or presence of 250, 500, 1000 µM GSH, in a vertical orientation. Root length of tobacco seedlings was measured after 21 days.

(iii) Seeds from both WT and homozygous PCS1 lines, which overexpressed *NtPCS1*, were germinated on foam sheets. After 28 days, the seedlings were transferred to holes in a plastic septum placed in blue boxes, allowing only the roots to be immersed in a 1/2 Hoagland solution. Subsequently, after 12 days, the tobacco seedlings were moved to a fresh solution containing 60 µM CdCl_2_, with or without the presence of 1000 µM GSH. The hydroponic solution was refreshed every 2 to 3 days. After 14 days, samples of shoots and roots were collected for HPLC (1200LC/MS, Varian) and atomic absorption spectroscopy analysis (AAS) (AA 700, PerkinElmer).

### *A. thaliana Atpcs1* mutant complementation

The *A. thaliana Atpcs1* mutant was grown in the soil. After 30 days, plants were transformed with *A. tumefaciens* strain *GV3101* harboring one of six different plant gene expression constructs (pBI121-*AtPCS1pro::NtPCS1-F*, pBI121-*AtPCS1pro::NtPCS1-N*, pBI121*-AtPCS1pro::NtPCS1-C*, pBI121-*35Spro::NtPCS1-F*, pBI121-*35Spro::NtPCS1-N*, and pBI121-*35Spro::NtPCS1-C*). These constructs contained either the full length of *NtPCS1* (*NtPCS1-F*, 1-501 aa), the N-terminal region of *NtPCS1* (*NtPCS1-N*, 1-219 aa) or the C-terminal region of *NtPCS1* (*NtPCS1-C*, 220-501 aa) under the control of 2,020 bp *AtPCS1* promoter or the CaMV35S promoter, respectively. Transgenic seeds were selected on 1/2 MS medium containing 30 mg/L kanamycin. Homozygous transgenic seeds were germinated and grown on 1/2 MS medium containing 0, 10, 20, 30, 40 or 50 µM CdCl_2_ in a vertical orientation. Root length was measured after 9 days.

### Elements analysis

Shoots and roots from tobacco treated with CdCl_2_ were separately harvested and dried at 75°C until a constant weight was reached. Dried samples were weighed and ground into powder. A 100 mg of powder material was digested for 2 hours in concentrated HNO3 at 220°C, and then the sample diluted with deionized water a concentration of less than 1% HNO3. The diluted sample was sent to the analytical testing center platform of Southwest University of Science and Technology, where the concentration of Cd^2+^ in the sample was determined using AAS (AA 700, PerkinElmer) analysis.

Samples are converted into atomic vapor at high temperatures using a flame and graphite furnace atomization system. The characteristic radiation of the samples is then emitted by the hollow cathode lamp specific for Cd and combined with the atomic vapor of Cd, resulting in spectral absorption reactions. Different spectral graphs are formed based on the Cd concentration and intensity in the samples. After passing through the instrument’s optical path analysis system, the optical-electrical converter, and the circuit system, the computer collects and analyzes the information data, ultimately outputting the analysis results. The analytical results were validated against a blank solution of 1M HNO3. Calibration curves were established using standard Cd solutions, including solutions with concentrations of 0, 0.1, 0.2, 0.4, 1, and 2 ng/ml.

### Cys, GSH and PC analysis

The shoots and roots of tobacco treated with CdCl_2_ were collected separately. A 100 mg plant samples were derivatized with monobromobimane (mBBr) and used for Cys, GSH, PC2, PC3 and PC4 analysis by HPLC (1200LC/MS, Varian) as previously described (Sneller et al., 2000).

### Bioinformatic analysis

The CDS (sequence ID: mRNA_24867_cds) and genomic DNA sequence (sequence ID: Ntab-BX_AWOK-SS1311) of the *NtPCS1* gene were obtained from the Sol Genomics Network (https://solgenomics.net/). Amino acid sequences of PCS proteins from various species are retrieved from the NCBI database (https://www.ncbi.nlm.nih.gov/). Multiple PCS protein sequence alignment was performed using the DNAMAN 7 software with the default parameters. For phylogenetic analysis, a neighbor-joining tree (bootstrap method 1000) was drawn with MEGA 6 software. Sequence identity of PCS protein sequences was determined by the BioEdit software. The physicochemical properties of the NtPCS1 protein were predicted using the ProtParam website (http://web.expasy.org/protparam/). Domains of the NtPCS1 protein were analyzed online using the InterPro database (http://www.ebi.ac.uk/interpro/scan.html). DNA regulatory motifs and elements were predicted using the online databases PLACE (https://place.com/) and PlantCARE (http://bioinformatics.psb.ugent.be/webtools/plantcare/html/), as described by Higo et al. (1999) and Lescot et al. (2002) (Higo et al., 1999; Lescot et al., 2002).

## Results

### Sequence analysis of NtPCS1

The *NtPCS1* gene encodes a 501 amino acids polypeptide with a predicted molecular weight of 55 kDa. The sequence alignment of PCS proteins from different species showed that the N-terminal region of PCS proteins was highly conserved (**Supplementary Figure S1**), and shared two N-terminal conserved motifs with the consensus sequence [T-Q-x-E-P-A-[YF]-C-G-L-x(2)-L-x(3)-L-N-[AS]-L-x(2)-D-P-x(3)-W-K-[GA]-[PS]-W-R-x(5)-M-L-D-C-C] and [Q-T-G-x-G-H-F-S-P-x(11)-L-I-[LM]-D-V-A-R-F-K-Y-P-[PC]-[HY]-W-V]. By analyzing the phylogenetic relationships among PCS proteins from different species, we found that NtPCS1 is most related to PCS proteins from solanaceae plants *Nicotiana rustica* (NrPCS), *Nicotiana glauca* (NgPCS) and *Solanum tuberosum* (StPCS1) because they were clustered into the same clade, shared approximately 98.6%, 96.4%, and 72.9% sequence similarity, respectively. Simultaneously, NtPCS1 is most distant to homologous proteins from *S. pombe* (SpPCS) and *C. elegans* (CePCS), with only about 4.9% and 5.1% sequence identity (**Supplementary Figure S2**; **Supplementary Table S1**).

### Enzyme activity of NtPCS1

**Figure 1:**
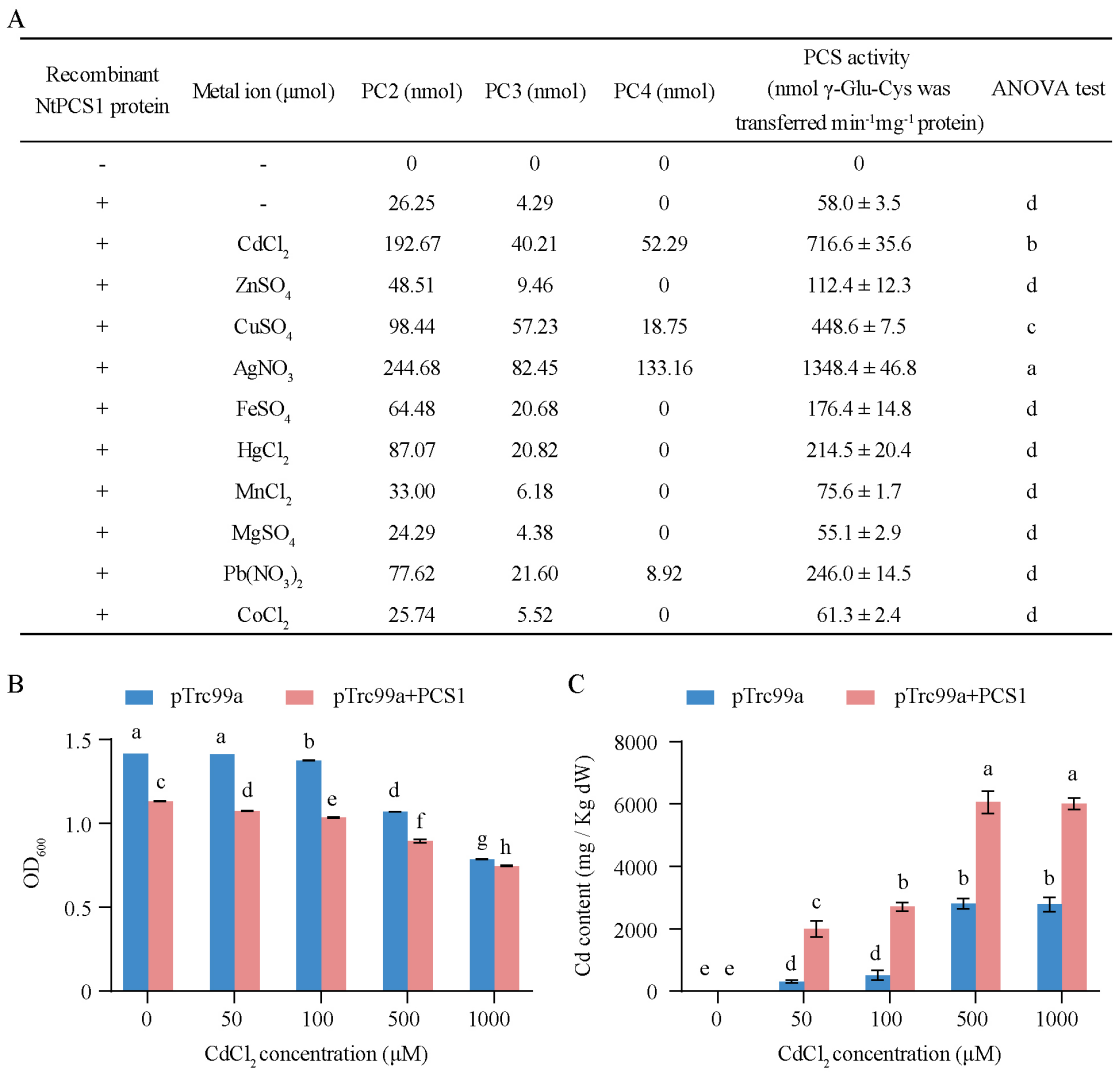
Enzyme activity of the NtPCS1 protein. **(A)** A 30 µg of purified recombinant NtPCS1 protein from *E. coli* and 100 µM various metal ions were used for PCS activity. PCs content was analyzed by HPLC. The PCS activity is expressed as the total nmols of γ - Glu - Cys transferred per minute per mg of the NtPCS1 protein. “-” in the first and second columns indicates the absence of the recombinant NtPCS1 protein and metal ions, respectively. “+” indicates the addition of the recombinant NtPCS1 protein. Values represent means ± SD of three biological replicates. Different letters indicate statistically significant differences (one-way ANOVA followed by a Tukey’s HSD test, *P<* 0.05). **(B, C)** The Cd tolerance of *E. coli* bacterial cells bearing the empty pTrc99A vector or the *NtPCS1* constructs was assessed by monitoring OD_600_ value **(B)** and intracellular Cd content **(C)**. Bacteria were cultured at 37°C until OD_600_ reached 0.6, then 100 µM IPTG and different concentrations of CdCl_2_ (0, 50, 100, 500, 1000 µM) were added and further grown for 3 hours to measure OD_600_ value and intracellular Cd content. Bacterial cells transformed pTrc99A vector were used as a control. Values represent means ± SD of three biological replicates. Different letters indicate statistically significant differences (two-way ANOVA followed by a Tukey’s HSD test, *P<* 0.05).

To confirm that the *NtPCS1* gene encodes phytochelatin synthase activity, the CDS was ligated into the pET28a vector and expressed in *E. coli*. The recombinant NtPCS1 protein was purified and used for enzyme activity test. The results revealed that without the recombinant NtPCS1 protein, no phytochelatin synthase activity was observed (**Figure 1A**). In the presence of the recombinant NtPCS1 protein, the synthesis of PC2 and PC3 was detected (**Figure 1A**). This suggests that the recombinant NtPCS1 protein has the ability to catalyze the synthesis of PC2 and PC3 *in vitro*. Previous studies showed that the enzyme activity of the AtPCS1 protein was up-regulated by a wide group of heavy metals (Sharma et al., 2016; Seregin and Kozhevnikova, 2023). We found that various metals can also enhance the enzyme activity of the recombinant NtPCS1 protein *in vitro*. The activity was significantly enhanced by the metals Ag^2+^, Cd^2+^, and Cu^2+^ in that order and it was enhanced to some extent by Pb^2+^, Hg^2+^, Fe^2+^, and Zn^2+^ (**Figure 1A**). Notably, PCS activity was up to 23-fold and 12-fold higher in the present of Ag^2+^ or Cd^2+^ than condition without metals (**Figure 1A**). In addition, Mn^2+^, Co^2+^ and Mg^2+^ were unable to increase PCS activity (**Figure 1A**).

To explore the effect of CdCl_2_ treatment on the growth and metal accumulation of engineered bacterial cells expressing *NtPCS1*, the OD_600_ values and intracellular Cd content of engineered bacterial cells were measured after treatment with different levels of CdCl_2_. The experimental data indicated that the growth of engineered bacterial cells expressing *NtPCS1* was inhibited (**Figure 1B**), and the intracellular Cd content was increased at various CdCl_2_ concentrations compared with bacterial cells transformed with pTrc99A vector (**Figure 1C**).

### The *NtPCS1* gene is constitutively expressed

**Figure 2:**
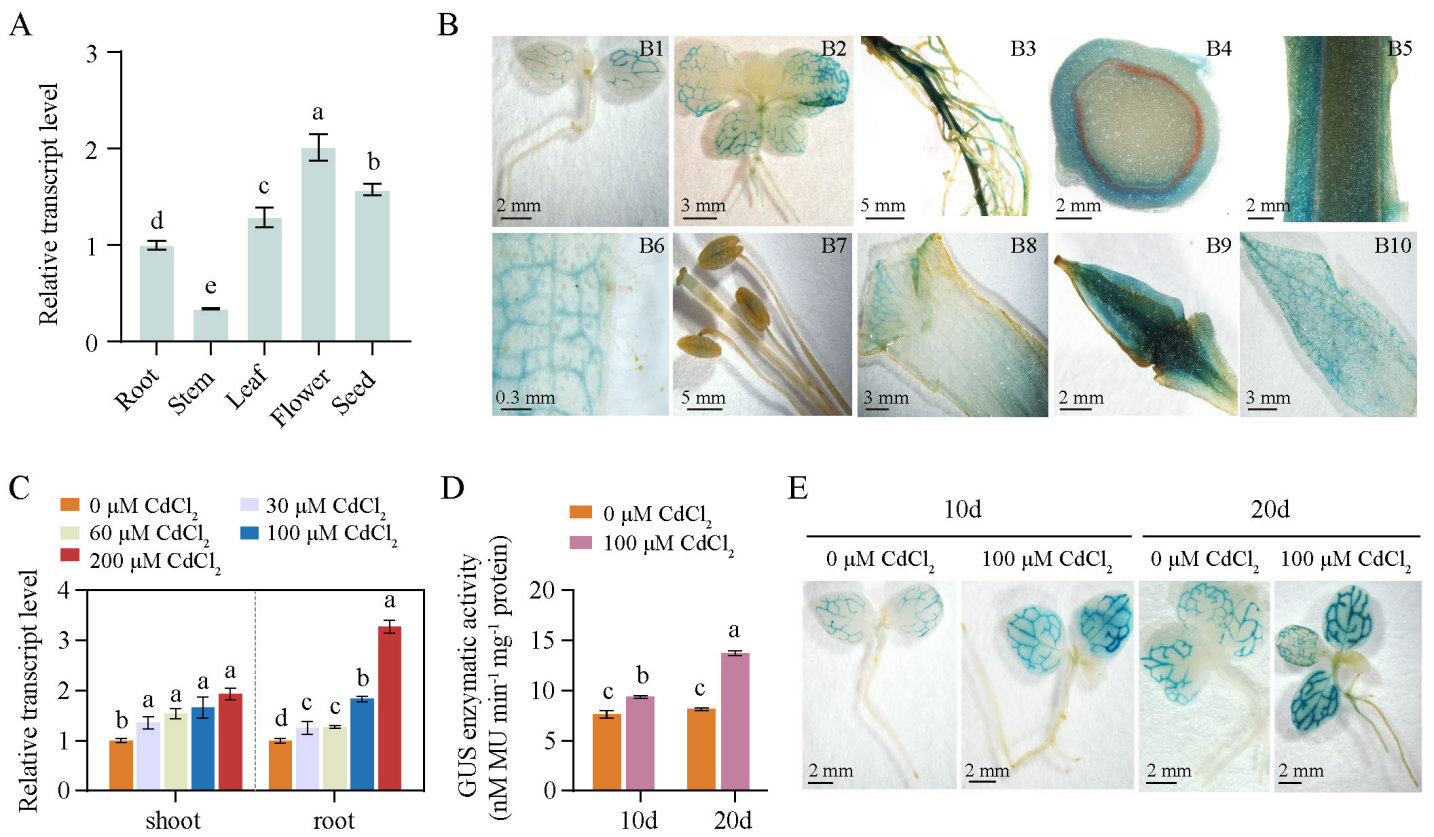
The expression pattern of the *NtPCS1* gene. **(A)** Transcript level of *NtPCS1* in different tissues of WT plants were detected by RT-PCR. Root, stem and leaf samples were collected from 40-day-old plants, flowers were obtained from 3-month-old plants during the flowering period, and seed were obtained from 5-month-old mature plants. Values represent means ± SD of three biological replicates. Different letters indicate statistically significant differences (one-way ANOVA followed by a Tukey’s HSD test, *P<* 0.05). **(B)** Histochemical staining of transgenic tobacco expressing pBI121*-NtPCS1pro::GUS*. Images depict 10-day-old seedlings (B1) and 1-month-old seedlings (B2), as well as root (B3), transverse section (B4) and longitudinal section (B5) of stem, leaf (B6), stigma and anthers (B7), petal (B8), ovary (B9), and sepal (B10) of 3-month-old plants. **(C)** Effect of various concentrations of CdCl_2_ (0, 30, 60, 100, 200 μM) on the transcript level of *NtPCS1* in the shoots and roots of WT plants was detected by RT-PCR. Values represent means ± SD of three biological replicates. Different letters indicate statistically significant differences (one-way ANOVA followed by a Tukey’s HSD test, *P<* 0.05). Separate statistical analyses were conducted for shoot and root samples. **(D, E)** Effect of 100 μM CdCl_2_ on the transcription activation of the *GUS* gene driven by the 1,097 bp promoter of *NtPCS1* was detected via GUS enzymatic activity **(D)** and staining **(E)** assays. Transgenic tobacco seeds expressing pBI121*-NtPCS1pro::GUS* were germinated and grown on 1/2 MS medium supplemented with 0 or 100 µM CdCl_2_. After 10 and 20 days, seedlings were harvested for GUS enzymatic activity and staining assays. Values represent means ± SD of three biological replicates. Different letters indicate statistically significant differences (two-way ANOVA followed by a Tukey’s HSD test, *P<* 0.05). Three independent transgenic tobacco lines expressing pBI121*-NtPCS1pro::GUS* were used in **(B, D, E)**, and similar results were obtained.

To investigate whether the *NtPCS1* gene is constitutively expressed, we analyzed the transcript levels of *NtPCS1* in different tissues of WT using RT-PCR, including root, stem, leaf, flower, and seed. The results indicated that the transcript level of *NtPCS1* was detected in all the tissues tested, with the order of expression effectiveness being flower > seed > leaf > root > stem (**Figure 2A**). To obtain more information on *NtPCS1* gene expression, we analyzed tobacco transformed with a GUS reporter driven by 1,097 bp upstream of the *NtPCS1* ATG start codon. Based on histochemical staining, GUS activity was detected in leaf veins of 10-day-old seedlings (**Figure 2B**, picture **Figure 2B1**). In 1-month-old seedlings, GUS activity was observed in leaf veins of both growing and fully expanded leaves, as well as in vascular tissues of young roots, but not detectable in new leaves, petiole, young stem, and root hairs (**Figure 2B**, picture **Figure 2B2**). In 3-month-old mature plants, GUS staining was observed in mature roots (**Figure 2B**, picture **Figure 2B3**) and in the epidermal cells of mature stems (**Figure 2B**, pictures **Figures 2B4, B5**). GUS expression was also detected in leaf veins of mature leaves, but not in leaf trichomes (**Figure 2B**, picture **Figure 2B6**). The reproductive organs displayed variable levels of GUS activity, with low GUS activity observed in stigma and anthers (**Figure 2B**, picture **Figure 2B7**), moderate GUS activity was detected in petals (**Figure 2B**, picture **Figure 2B8**), and ovary (**Figure 2B**, picture **Figure 2B9**) and sepals (**Figure 2B**, picture **Figure 2B10**) showed strong GUS staining. These results are consistent with RT-PCR data, suggesting that *NtPCS1* is constitutively expressed.

### Cd induces an increase of *NtPCS1* transcription

The mRNA expression level of *AtPCS1* was not regulated by Cd treatment in *A. thaliana* (Lee et al., 2002), while the BjPCS protein was increased significantly in *B. juncea* leaves after prolonged Cd exposure (Heiss et al., 2003). To investigate the effect of heavy metals on the expression of the *NtPCS1* gene in tobacco, we treated 35-day-old WT seedlings with various concentrations of CdCl_2_ (0, 30, 60, 100, 200 μM) for 48 hours and then conducted RT-PCR to detect the transcript level of *NtPCS1*. The data showed that the transcript level of *NtPCS1* were up-regulated by CdCl_2_ in tobacco (**Figure 2C**). Under 200 μM CdCl_2_, the transcript level of *NtPCS1* were up-regulated to 1.94- and 3.27-fold in shoots and roots, respectively (**Figure 2C**). These results indicate that the expression of *NtPCS1* is regulated by Cd stress. In addition, we analyzed the time-course response of *NtPCS1* to CdCl_2_ treatment. The 35-day-old WT seedlings were cultivated in solution containing 0 or 60 μM CdCl_2_, and grown for additional 3, 5, and 7 days. Transcript level of *NtPCS1* in shoots were detected using RT-PCR. The experimental result showed that CdCl_2_ treatment induced upregulation of *NtPCS1* expression at all detected time points, with a significant increase at 5 days compared to 3 days, and a slight additional increase at 7 days that was not statistically significant when compared to 5 days (**Supplementary Figure S3**). These results further confirm that the expression of *NtPCS1* is regulated by Cd.

To gain further information on the transcriptional regulation of *NtPCS1*, seeds of transgenic tobacco expressing pBI121-*NtPCS1pro::GUS* construct were germinated and grown on 1/2 MS medium containing 100 μM CdCl_2_. After 10 and 20 days, seedlings were collected for GUS enzymatic activity analysis. We found that CdCl_2_ induced a higher GUS enzyme activity and a stronger GUS staining compared to control (**Figures 2D, E**). All the above experimental results indicated that the transcript level of the *NtPCS1* gene was up-regulated by CdCl_2_.

Using the PLACE and PlantCARE databases (Higo et al., 1999; Lescot et al., 2002), we identified that the promoter region of *NtPCS1* contains various typical DNA regulatory motifs, including the CAAT box and TATA box, as well as several light-responsive elements such as the ACE, AE box, ATCT motif, Box 4, Box I, GATA motif, I box, and Sp1 (**Supplementary Table S2**). Meanwhile, there are two cis-regulatory elements GCN4 motif and Skn-1 motif which are involved in endosperm expression (**Supplementary Table S2**). Moreover, the MBS element related to drought-inducibility, WUN-motif associated with wound-responsive, and TC-rich repeats involved in defense and stress responsiveness were found (**Supplementary Table S2**). Because the *AtPCS1* expression is not regulated by Cd treatment (Lee et al., 2002), we compared the 1,097 bp promoter region of *AtPCS1* with *NtPCS1* promoter. Interestingly, although both two promoters shared several regulatory motifs, the *AtPCS1* promoter region harbored a set of unique regulatory elements. These include light-responsive elements such as AAAC, ATC motif, G-box, GAG motif, GT1 motif, and LAMP element, as well as Box-W1 for fungal elicitor response, CGTCA motif and TGACG motif for MeJA responsiveness, LTR for low-temperature responsiveness, ARE which is crucial for anaerobic induction, MBSII for flavonoid biosynthetic gene regulation, Circadian element for circadian control, and W box, which function remains unknown (**Supplementary Table S2**). However, several regulatory elements, including ACE, AE box, ATCT motif, GATA motif, I box, Sp1, GCN4 motif, MBS element, and TC-rich repeats, were present in the *NtPCS1* promoter region but absent in the *AtPCS1* promoter (**Supplementary Table S2**). Given that the MBS element and TC-rich repeats are involved in stress responsiveness (Zhao et al., 2023), we speculate that they may play an important role in the metal-mediated regulation of *NtPCS1* transcription expression, leading to the difference between *NtPCS1* and *AtPCS1* in response to Cd stress.

### Overexpression of *NtPCS1* in tobacco results in increased sensitivity to Cd, which can be alleviated by the addition of GSH

**Figure 3:**
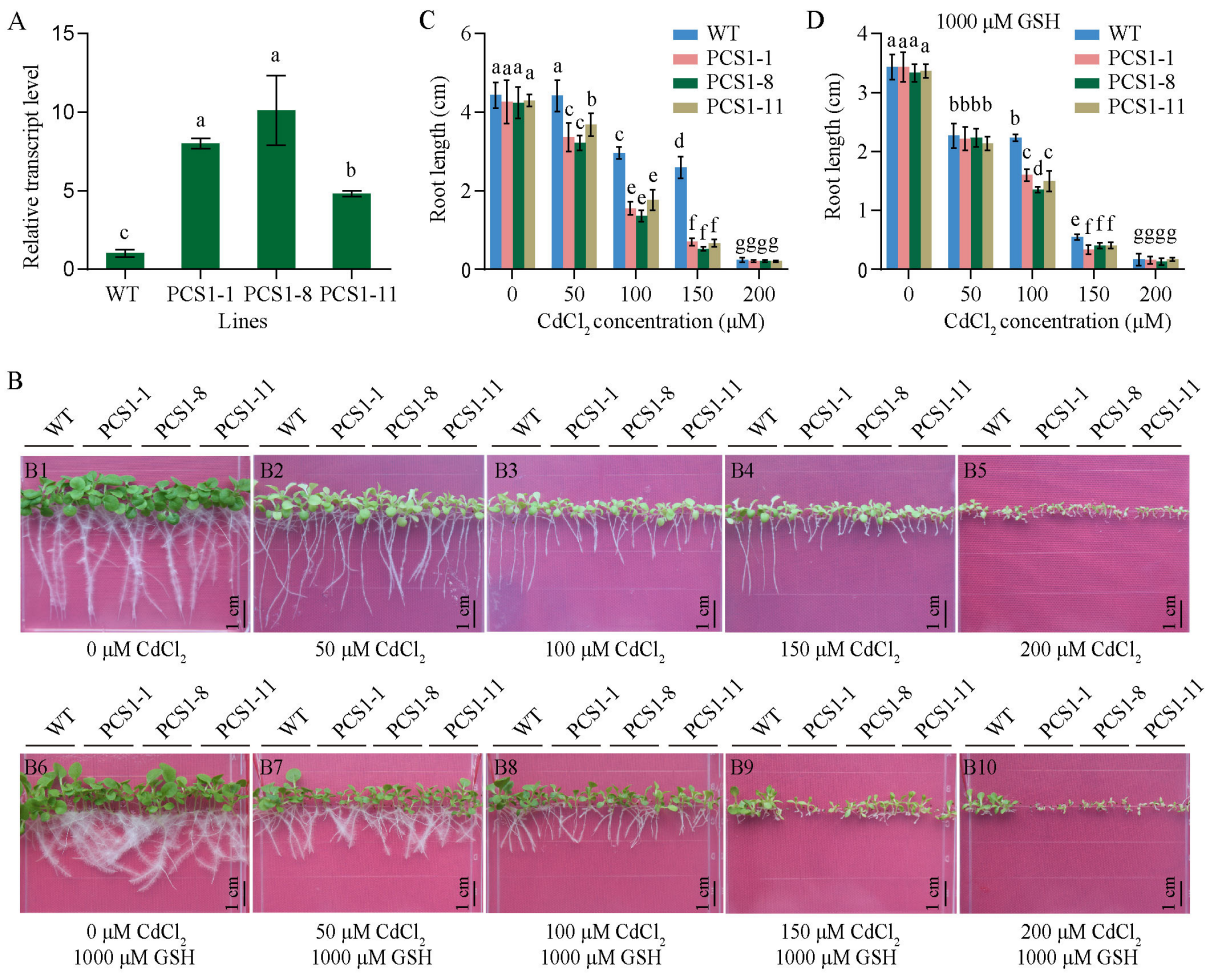
Phenotypes of PCS1 lines under CdCl_2_ stress. **(A)** Transcript level of *NtPCS1* in the WT and PCS1 lines were detected by RT-PCR. The PCS1 lines refers to the transgenic tobacco transformed with the pBI121-*35Spro::NtPCS1* construct. Values represent means ± SD of three biological replicates. Different letters indicate statistically significant differences (one-way ANOVA followed by a Tukey’s HSD test, *P<* 0.05). **(B)** Phenotype of WT and PCS1 lines under different concentrations of CdCl_2_ with or without GSH, encompassing: 0 (B1), 50 (B2), 100 (B3), 150 (B4), and 200 (B5) μM CdCl_2_ in the absence of 1000 μM GSH, as well as 0 (B6), 50 (B7), 100 (B8), 150 (B9), and 200 (B10) μM CdCl_2_ in the presence of 1000 μM GSH. **(C, D)** Root length of WT and PCS1 lines under various concentrations of CdCl_2_ (0, 50, 100, 150, 200 μM) in the absence **(C)** and presence **(D)** of 1000 μM GSH. Tobacco seeds were germinated and grown in a vertical orientation on 1/2 MS medium containing CdCl_2_ with and without GSH. After 21 days, the root length of WT and PCS1 lines was measured. Values represent means ± SD (n = 24). Different letters indicate statistically significant differences (two-way ANOVA followed by a Tukey’s HSD test, *P<* 0.05).

Transgenic tobacco transformed with pBI121-*35Spro::NtPCS1* construct was named PCS1 lines. The transcript level of *NtPCS1* was detected using RT-PCR. PCS1 lines (PCS1-1, PCS1-8, PCS1-11) exhibited higher expression than WT, with approximately 8-, 10-, and 4.8-fold increase, respectively (**Figure 3A**). To evaluate Cd tolerance of PCS1 lines and WT, seeds were plated on 1/2 MS medium containing various concentrations of CdCl_2_ (0, 50, 100, 150, 200 μM). The root length was measured after 21 days. When seeds were grown on 0 μM CdCl_2_, all seedlings grew normally and no significant difference was observed between them (**Figure 3B**, picture **Figure 3B1**; **Figure 3C**). In the presence of 50, 100, and 150 μM CdCl_2_, growth of tobacco seedlings was affected, with serious chlorosis (**Figure 3B**, pictures **Figures 3B2–B4**). Meanwhile, PCS1 lines showed more sensitive to CdCl_2_ than WT (**Figures 3B2–B4**; **Figure 3C**). When CdCl_2_ concentration was increased to 200 μM, the growth of all tobacco seedlings was almost completely inhibited (**Figure 3B**, picture **Figure 3B5**; **Figure 3C**).

Tobacco overexpressing *AtPCS1* was more sensitive to Cd than control (Wojas et al., 2008), whereas it exhibited increased Cd tolerance and accumulation, when exogenous GSH was added to the growth medium (Pomponi et al., 2006). Thus, the effects of GSH on the response to Cd were assessed. In the presence of 250, 500 or 1000 μM GSH, no significant difference was observed between PCS1 lines and WT under 0 μM CdCl_2_ (**Figure 3B**, picture B6; **Figure 3D**; **Supplementary Figures S4A, B**). Interestingly, the difference in root growth between PCS1 lines and WT, which was observed under 50 μM CdCl_2_ without GSH, gradually decreased and even almost completely disappeared under 50 μM CdCl_2_ and 1000 μM GSH (**Figure 3B**, picture B7; **Figure 3D**). Furthermore, under 100, 150, and 200 μM CdCl_2_ in the presence of 250, 500, or 1000 μM GSH, the pattern of root growth was similar to that observed in the absence of GSH, with the PCS1 lines exhibiting greater sensitivity to CdCl_2_ than the WT (**Figure 3B**, pictures **Figures 3B3, B4, B8, B9**; **Figure 3D**; **Supplementary Figures S4A, B**) at 100 and 150 μM, and the root growth of all tobacco seedlings was almost completely inhibited (**Figure 3B**, picture **Figure 3B10**; **Figure 3D**; **Supplementary Figures S4A, B**) at 200 μM CdCl_2_.

### Heterologous expression of *NtPCS1* results in enhanced Cd tolerance in *A. thaliana Atpcs1* mutant

**Figure 4:**
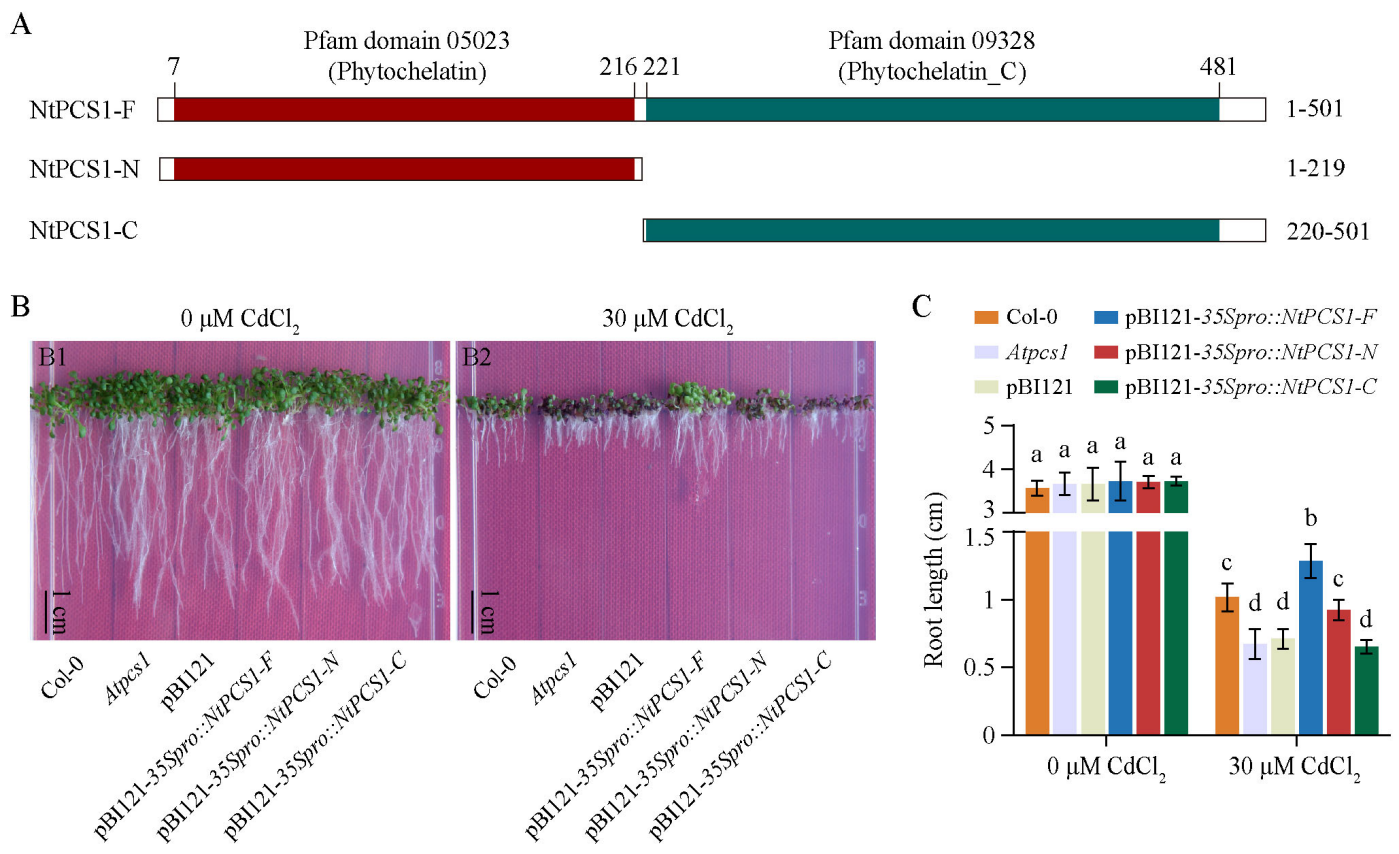
Functional complementation of the *A. thaliana Atpcs1* mutant by heterologous expression of *NtPCS1*. **(A)** Schematic diagram of full-length and truncated versions of the NtPCS1 protein. Amino acid positions of truncated protein are labelled. The phytochelatin and phytochelatin_C domains in the NtPCS1 protein are highlighted in red and green shadow, respectively. **(B, C)** Phenotypes **(B)** and root length **(C)** of Col-0, *Atpcs1* mutant and transgenic *A. thaliana* seedlings expressing pBI121-*35Spro::NtPCS1-F/N/C* under CdCl_2_ stress. *A. thaliana* seeds were germinated and grown in a vertical orientation on 1/2 MS medium containing 30 μM CdCl_2_. Root length was measured after 9 days. NtPCS1-F represents the full-length of NtPCS1 (1-501 aa), NtPCS1-N represents the N-terminal region of NtPCS1(1-219 aa), and NtPCS1-C represents the C-terminal region of NtPCS1 (220-501 aa). Col-0: Columbia-0; *Atpcs1*: *Atpcs1* mutant; pBI121: *Atpcs1* mutant transformed with empty pBI121 vector; pBI121-*35Spro::NtPCS1-F/N/C*: *Atpcs1* mutant transformed with pBI121-*35Spro::NtPCS1-F/N/C*, respectively. Values represent means ± SD (n = 24). Different letters indicate statistically significant differences (two-way ANOVA followed by a Tukey’s HSD test, *P<* 0.05). Three independent transgenic *A. thaliana* lines were used in **(B, C)**, and similar results were obtained. Shown are the experimental results for one of the three transgenic *A. thaliana* lines.

To explore the essential active site of the NtPCS1 protein, we predicted the protein domain using the InterPro online analysis tool. The schematic diagram of the protein domains was generated using the IBS 1.0 software (Liu et al., 2015). The NtPCS1 protein was found to possess the Pfam domains 05023 (Phytochelatin) and 09328 (Phytochelatin_C), which are typical for PCS proteins in higher plants. Consequently, the NtPCS1 protein was divided into two parts, named NtPCS1-N and NtPCS1-C, representing the two domains, and the full length of NtPCS1 was named NtPCS1-F (**Figure 4A**).

To investigate the function of different parts of the NtPCS1 protein, the *A. thaliana Atpcs1* mutant was transformed with *NtPCS1-F*, *NtPCS1-N* or *NtPCS1-C* under the control of the *AtPCS1* promoter or the CaMV35S promoter (**Supplementary Figure S5A**). The *A. thaliana* seeds were germinated and grown in a vertical orientation on 1/2 MS medium containing various concentrations of CdCl_2_ (0, 10, 20, 30, 40, and 50 μM). When seeds were grown on 0 μM CdCl_2_, the WT (Col-0), *Atpcs1* mutant, and transgenic seedlings grew normally and no significant difference was observed between them (**Figure 4B**, picture **Figure 4B1**; **Figure 4C**; **Supplementary Figure S5B**, picture **Supplementary Figure S5B1**; **Supplementary Figure S5C**). In the presence of 10, 20, 30, 40, and 50 μM CdCl_2_, the *Atpcs1* mutant displayed more sensitive to CdCl_2_ than WT (**Figure 4B**, picture **Figure 4B2**; **Figure 4C**; **Supplementary Figure S5B**, pictures **Supplementary Figures S5B2–B6**; **Supplementary Figure S5C**). When the *Atpcs1* mutant was transformed with pBI121-*AtPCS1pro::NtPCS1-F*, pBI121-*35Spro::NtPCS1-F*, and pBI121*-35Spro::NtPCS1-N* constructs, an improvement in root growth was observed compared to the *Atpcs1* mutant under 30 µM CdCl_2_ (**Figure 4B**, picture **Figure 4B2**; **Figure 4C**; **Supplementary Figure S5B**, picture **Supplementary Figure S5B4**; **Supplementary Figure S5C**). However, transformation with pBI121 vector, pBI121*-AtPCS1pro::NtPCS1-N*, pBI121-*AtPCS1pro::NtPCS1-C*, and pBI121-*35Spro::NtPCS1-C* showed no significant difference under 30 µM CdCl_2_ (**Figure 4B**, picture **Figure 4B2**; **Figure 4C**; **Supplementary Figure S5B**, picture **Supplementary Figure S5B4**; **Supplementary Figure S5C**). This suggests that the conserved N-terminal domains of NtPCS1 may play a crucial role in Cd tolerance. Furthermore, the transformation with pBI121-*35Spro::NtPCS1-F* completely complemented the Cd hypersensitivity of the *Atpcs1* mutant, and the transgenic seedlings even demonstrated enhanced root growth compared to the WT (**Figures 4B, C**), indicating that the heterologous expression of *NtPCS1* leads to enhanced Cd tolerance. In addition, this result strongly indicated that Cd hypersensitivity observed in PCS1 lines tobacco was not due to toxicity of the overexpressed NtPCS1 protein itself.

### Overexpression of *NtPCS1* results in increased PCs and Cd accumulation under Cd stress

**Figure 5:**
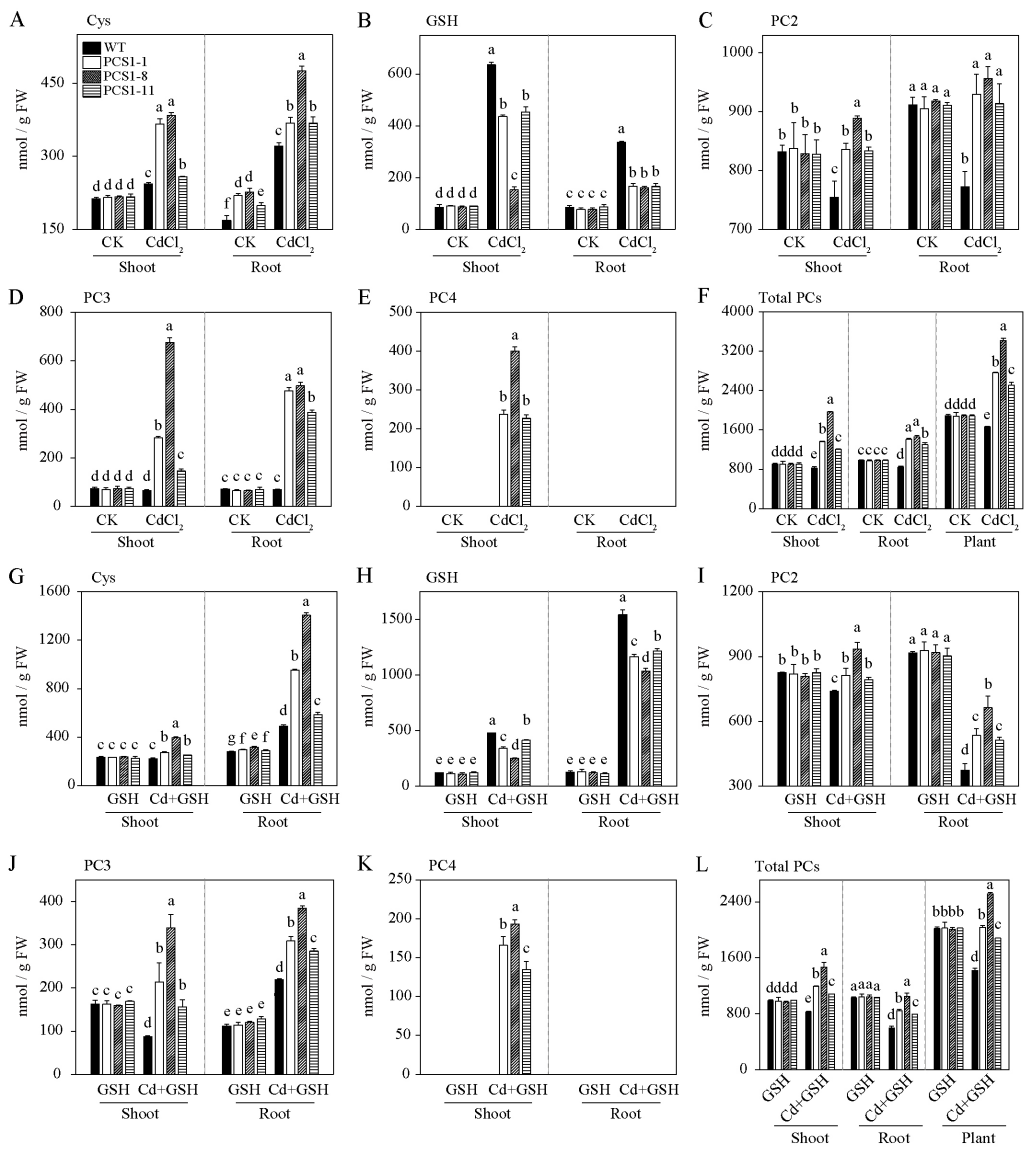
PCs content of WT and PCS1 lines under Cd stress. **(A–L)** The content of Cys, GSH, PC2, PC3, and PC4 in WT and PCS1 lines was analyzed by HPLC. Cys **(A)**, GSH **(B)**, PC2 **(C)**, PC3 **(D)**, PC4 **(E)**, and total PCs **(F)** were measured in WT and PCS1 lines under 0 and 60µM CdCl_2_. Similarly, Cys **(G)**, GSH **(H)**, PC2 **(I)**, PC3 **(J)**, PC4 **(K)**, and total PCs **(L)** were analyzed in WT and PCS1 lines under 0 and 60µM CdCl_2_ in the presence of 1000 µM GSH. The 40-day-old hydroponically grown tobacco were incubated on 1/2 Hoagland solution containing 0 or 60 µM CdCl_2_ in the absence or presence of 1000 µM GSH. After 14 days, shoots and roots were harvested for HPLC analysis. Total PCs is equal to the sum of PC2, PC3 and PC4. The PCS1 lines refers to the transgenic tobacco transformed with the pBI121-*35Spro::NtPCS1* construct. CK, control, 0 µM CdCl_2_; CdCl_2_: 60 µM CdCl_2_; GSH: 1000 µM GSH; Cd + GSH: 60 µM CdCl_2_ + 1000 µM GSH. Values represent means ± SD of three biological replicates. Different letters indicate statistically significant differences (two-way ANOVA followed by a Tukey’s HSD test, *P<* 0.05). Separate statistical analyses were conducted for shoot, root, and plant samples.

To investigate the effects of overexpression of *NtPCS1* on PCs and metal accumulation, 40-day-old WT and PCS1 lines tobacco were exposed to 60 µM CdCl_2_ in the absence or presence of 1000 µM GSH in a hydroponics seedling assay. Shoots and roots were separately harvested after 14 days. Cys, GSH, PC2, PC3 and PC4 contents were detected by HPLC. Under control without metal, Cys contents of PCS1 lines were not significantly different from WT in shoots, but these were much higher than WT in roots (**Figure 5A**). Meanwhile, GSH contents of PCS1 lines were comparable to WT in both shoots and roots (**Figure 5B**). When tobacco was subjected to 60 µM CdCl_2_, Cys and GSH contents were increased in both PCS1 lines and WT compared to control (**Figures 5A, B**). Cys concentrations in PCS1 lines were substantially higher than those in WT, but GSH concentrations of PCS1 lines were less than WT (**Figures 5A, B**). To further investigate whether decreased GSH contents actually reflected PC contents, PC2, PC3, PC4 and total PCs content were measured. In summary, PC2, PC3, PC4, and total PCs in the shoots of PCS1 lines were significantly increased compared with WT, and PC2, PC3, and total PCs in the roots of PCS1 lines were also significantly increased compared with WT under Cd stress (**Figures 5C–F**). Total PCs of PCS1 lines was increased to 1.5 to 2.0-fold compared to WT in whole plant (**Figure 5F**). In addition, no PC4 was detected in the WT under all conditions (**Figure 5E**). When plants were grown in solution supplied with 1000 µM exogenous GSH, Cys, GSH, PC2, PC3, PC4, and total PCs contents of WT and PCS1 lines displayed similar trend compared with condition without GSH (**Figures 5A–L**). Notably, under Cd stress, PCS1 lines exhibited higher levels of Cys, PC2, PC3, and total PCs in both shoots and roots, as well as a higher PC4 content in shoots, compared to the WT (**Figures 5G–L**).

**Figure 6:**
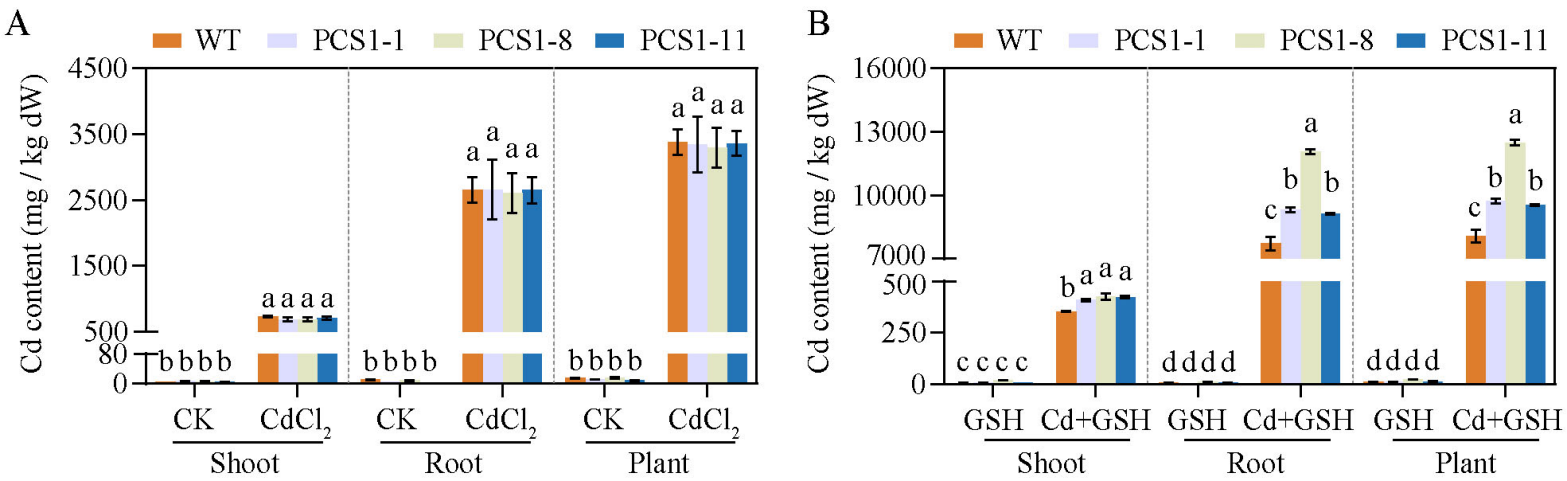
Cd accumulation of WT and PCS1 lines under Cd stress. **(A, B)** Cd content of WT and PCS1 lines under 0 and 60µM CdCl_2_ in the absence **(A)** or presence **(B)** of 1000 µM GSH. The 40-day-old hydroponically grown tobacco were incubated on 1/2 Hoagland solution containing 0 or 60 µM CdCl_2_ in the absence or presence of 1000 µM GSH. Shoots and roots were harvested after 14 days. The Cd content was analyzed by AAS. The PCS1 lines refers to the transgenic tobacco transformed with the pBI121-*35Spro::NtPCS1* construct. CK, control, 0 µM CdCl_2_; CdCl_2_: 60 µM CdCl_2_; GSH: 1000 µM GSH; Cd + GSH: 60 µM CdCl_2_ + 1000 µM GSH. Values represent means ± SD of three biological replicates. Different letters indicate statistically significant differences (two-way ANOVA followed by a Tukey’s HSD test, *P<* 0.05). Separate statistical analyses were conducted for shoot, root, and plant samples.

The Cd content of WT and PCS1 lines was analyzed by AAS. In summary, tobacco plants overexpressing *NtPCS1* exhibited increased Cd accumulation when the plants were grown in a solution supplemented with both CdCl_2_ and exogenous GSH (**Figures 6A, B**). Minimal Cd was detected in tobacco grown under control conditions or with 1000 µM GSH (**Figures 6A, B**). However, under CdCl_2_ stress, substantial Cd accumulation was observed in both shoots and roots, with the roots showing higher Cd concentrations than the shoots (**Figures 6A, B**). When plants were exposed to 60 μM CdCl_2_, there were no differences in Cd accumulation between the PCS1 lines and the WT in both shoots and roots (**Figure 6A**). Interestingly, the addition of 1000 µM exogenous GSH along with 60 μM CdCl_2_ to the hydroponic solution led to a dramatic increase in Cd concentrations in the roots of all tobacco, as compared to the condition without GSH (**Figures 6A, B**). Furthermore, the PCS1 lines accumulated more Cd than the WT in both shoots and roots when exposed to 60 µM CdCl_2_ and 1000 µM exogenous GSH (**Figures 6A, B**).

## Discussion

PCs are crucial for detoxification or the maintenance of metal ion homeostasis (Rea, 2012; Seregin and Kozhevnikova, 2023). *PCS* and *PCS*-like genes have been identified in various species, including yeast, animals and plants (Seregin and Kozhevnikova, 2023). Overexpression of the *PCS* gene has been demonstrated to enhance tolerance and accumulation of Cd, Zn, Pb, and As in transgenic plants, yeast, and even bacteria (Vatamaniuk et al., 1999; Sauge-Merle et al., 2003; Pomponi et al., 2006; Liu et al., 2011; Kuhnlenz et al., 2015; Zanella et al., 2016; Fan et al., 2018; Liu et al., 2021; Zhu et al., 2021). However, some studies have reported that *PCS* transgenic plants exhibit reduced tolerance to metals (Lee et al., 2003a, 2003b; Li et al., 2004; Wojas et al., 2008; Wang et al., 2012; Park et al., 2019). Although previous studies have shown that overexpression of *NtPCS1* in *S. cerevisiae* and tobacco can enhance tolerance to Cd and As (Kim et al., 2005; Lee and Hwang, 2015), detailed information about *NtPCS1* remains limited. This study aims to comprehensively investigate the molecular and expression characteristics, as well as the biological function of the *NtPCS1* gene, with a specific focus on determining whether the overexpression of *NtPCS1* can enhance Cd tolerance across different species.

PCS is an enzyme which plays a role in catalyzing PCs synthesis from GSH (Cobbett, 2000; Rea, 2012; Seregin and Kozhevnikova, 2023). Previous studies have indicated that PCS activity is induced by exposure to heavy metals both *in vivo* and *in vitro* (Chen et al., 1997; Ha et al., 1999; Heiss et al., 2003; Degola et al., 2014). In this study, under the control of no metal ions, enzyme activity of NtPCS1 protein was detected *in vitro* (**Figure 1A**). We speculate that these results may be due to interspecies differences or differences in experimental conditions. Moreover, the activity of PCS proteins has been shown to be stimulated by a range of heavy metal ions, including Cd^2+^, Ag^2+^, Cu^2+^, Au^2+^, Zn^2+^, Fe^2+^, Hg^2+^, As^3+^, and Pb^2+^, in various plant species such as *S. lycopersicum*, *Lunularia cruciata*, *A. thaliana*, and *B. juncea* (Chen et al., 1997; Ha et al., 1999; Heiss et al., 2003; Degola et al., 2014). Our data indicated that the activity of NtPCS1 was enhanced in the presence of Ag^2+^ > Cd^2+^ > Cu^2+^ > Pb^2+^ > Hg^2+^ > Fe^2+^ > Zn^2+^ (**Figure 1A**), which aligns well with previous findings in other species.

*PCS* genes from various plants, such as *A. thaliana* (Lee et al., 2002), *B. juncea* (Heiss et al., 2003), *Pyrus betulaefolia* (Chang et al., 2012), *O. sativa* (Cao et al., 2018), *Brassica parachinensis* (Fu et al., 2020), *Tagetes patula* (Zha et al., 2021), and *Salicornia europaea* (Zhu et al., 2022), are consistently expressed in different tissues. Expression analysis of the *NtPCS1* gene revealed a constitutive expression pattern in various tissues, including leaf veins, root vascular tissue, stem epidermal cells, ovary, sepals, and petals (**Figures 2A, B**). In contrast to the developmental suppression of *PCS* expression in *A. thaliana* siliques and tomato fruits (Chen et al., 1997; Lee et al., 2002), strong expression of the *NtPCS1* gene was observed in flowers and seeds of tobacco plants, indicating potential role in reproductive development. Given the diverse functions of PCS proteins in other species (Blum et al., 2007; Clay et al., 2009; Blum et al., 2010; Kuhnlenz et al., 2015; De Benedictis et al., 2018; Fontanini et al., 2018; Kim et al., 2019; Hématy et al., 2020; Inoue et al., 2022), the distribution of the *NtPCS1* gene in tobacco suggests that phytochelatin synthase may perform additional significant functions in plant physiological metabolic processes beyond metal detoxification. In addition, the regulation of *PCS* expression and activity plays a critical role in maintaining metal ion homeostasis. Studies have shown that *PCS* genes, including *BjPCS1* (Heiss et al., 2003; Ahmad and Gupta, 2013), *PbPCS1* (Chang et al., 2012), *MnPCS1/2* (Fan et al., 2018), *SoPCS* (Yousefi et al., 2018), *OsPCS5/7/9/15* (Park et al., 2019), and *SlPCS1* (Kısa, 2019), are upregulated by heavy metals, particularly Cd. Transcript level of *NtPCS1* increased notably in shoots and roots after Cd exposure, particularly in roots, supporting metal ion-mediated transcriptional regulation of *PCS* expression (**Figure 2C**).

The synthesis of PCs, catalyzed by γ-ECS, GS, and PCS, is closely associated with Cd tolerance. This connection is supported by findings such as the identification of *cad1*, a Cd-sensitive mutant of *A. thaliana* with impaired PC biosynthesis (Kuhnlenz et al., 2014, 2015), and the enhanced Cd tolerance observed in yeast strain with the overexpression of *AtPCS1* and *BrPCS* (Sauge-Merle et al., 2003; Liu et al., 2021). Overexpression of *PCS* genes is expected to increase PC levels and improve metal tolerance. However, the current study revealed that overexpression of *NtPCS1* in tobacco led to heightened sensitivity to Cd despite an increase in PCs production (**Figures 3B, C**, **5C–F**), different from the results of the previous study (Lee and Hwang, 2015). Given the significant variability in the responses of *PCS* genes from various species to metals, we hypothesized that this discrepancy might be due to differences between tobacco varieties. In addition, some studies have shown that *PCS*-overexpressing transgenic plants exhibit reduced tolerance to Cd and Zn (Lee et al., 2003a, 2003; Li et al., 2004; Wojas et al., 2008; Wang et al., 2012; Park et al., 2019). This suggests that PCs may not be the primary factor influencing Cd accumulation or tolerance in plants. It underscores that simply boosting PCs production may not be adequate for enhancing metal tolerance. This is in line with the understanding that AtPCS1 safeguards plants from heavy metal toxicity not only by synthesizing PCs but also by contributing to callose deposition (De Benedictis et al., 2018).

GSH serves as a direct substrate for PC synthesis. Increased Cd tolerance and accumulation were observed in tobacco overexpressing *AtPCS1* when supplemented with GSH in the growth medium (Pomponi et al., 2006). In this study, the impact of GSH on metal tolerance was evaluated by co-treating the medium with exogenous GSH and Cd. Exogenous GSH led to a gradual reduction in the difference between PCS1 lines and WT, and the discrepancy disappeared entirely under 1000 μM GSH and 50 μM CdCl_2_ (**Figures 3B, D**). Interestingly, the addition of exogenous GSH resulted in significantly enhanced Cd accumulation in PCS1 lines under Cd stress (**Figures 6A, B**), suggesting a direct link between GSH availability and metal accumulation. While the mechanism behind this effect could be related to the GSH-Cd complex as the rate-limiting substrate for PCS (Vatamaniuk et al., 2000), these results emphasize a direct relationship between GSH availability and Cd accumulation.

In addition, it was observed that heterologous expression of *CePCS* partially restored Cd hypersensitivity in the *A. thaliana cad1-3* mutant (Kuhnlenz et al., 2014, 2015). In this study, the pronounced Cd hypersensitivity of the *A. thaliana Atpcs1* mutant was partially alleviated by overexpressing the full length or N-terminal region of *NtPCS1* (**Figures 4B, C**). It is important to note that the heterologous overexpression of *NtPCS1* resulted in enhanced Cd tolerance compared to the WT, strongly suggesting that the Cd hypersensitivity observed in the PCS1 tobacco lines was not attributable to the toxicity of the overexpressed NtPCS1 protein. This study demonstrates that overexpression of *NtPCS1* in tobacco resulted in Cd hypersensitivity, whereas heterologous expression of *NtPCS1* in *A. thaliana* enhanced Cd tolerance. A similar phenomenon is observed in *A. thaliana*, where overexpression of *AtPCS1* in *A. thaliana* resulted in Cd hypersensitivity (Lee et al., 2003a, 2003; Li et al., 2004), while in *B. juncea*, it enhances tolerance to Cd, As, and Zn (Gasic and Korban, 2007a; 2007b). These findings together suggest that the effects of *PCS* gene overexpression or heterologous expression on metal tolerance can vary between species. In summary, this study not only profiles the expression of *NtPCS1* and PCS activity but also uncovers the biological role of the *NtPCS1* gene in Cd response across diverse species. This work enriches our understanding of the molecular aspects of the *NtPCS1* gene and suggests a promising avenue for enhancing metal tolerance through the heterologous expression of *PCS* genes in various species.

## Data Availability

The original contributions presented in the study are included in the article/**Supplementary Material**. Further inquiries can be directed to the corresponding author.
