# Supplementary material for: Characterization of a Nicotiana tabacum phytochelatin synthase 1 and its response to cadmium stress
Source: Front Plant Sci. 2024 Aug 29;15:1418762. doi: 10.3389/fpls.2024.1418762 (PMC11393743; doi:10.3389/fpls.2024.1418762)
Supplement: Supplementary file 1 [file DataSheet1.docx]

Supplementary Material


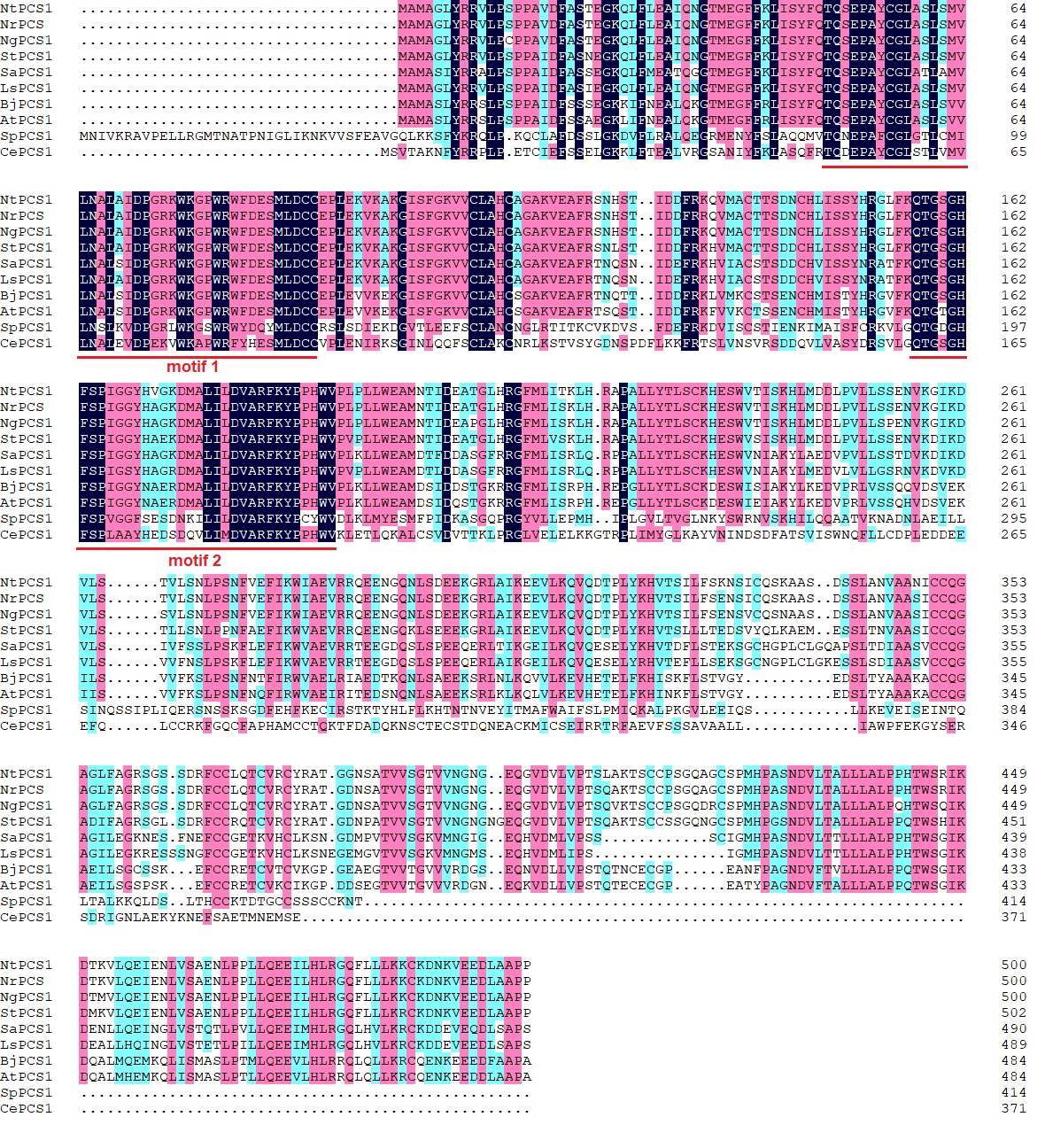


**Supplementary Figure 1.** Alignment of amino acid sequences of PCS proteins from different species. The alignment was performed using DNAMAN 7 software. Amino acid sequences of PCS proteins are retrieved from the NCBI database. The dark blue, pink, and blue-green shaded regions represent homology levels of amino acids at 100%, 75%, and 50%, respectively. Red lines indicate the N-terminal conserved motifs of PCS proteins. NtPCS1(*Nicotiana tabacum*, AAO74500.1), NrPCS (*Nicotiana rustica*, AJU57239.1), NgPCS1 (*Nicotiana glauca*, ABX10958.1), StPCS1 (*Solanum tuberosum*, CAD68110.1), SaPCS (*Sedum alfredii*, AHB86971.1), LjPCS1 (*Lotus japonicas*, AAQ01752.1), BjPCS1 (*Brassica juncea*, CAC37692.1), AtPCS1 (*Arabidopsis thaliana*, AAD50593.1), SpPCS (*Schizosaccharomyces pombe*, CAA92263.1), CePCS (*Caenorhabditis elegans*, AAK62992.1).


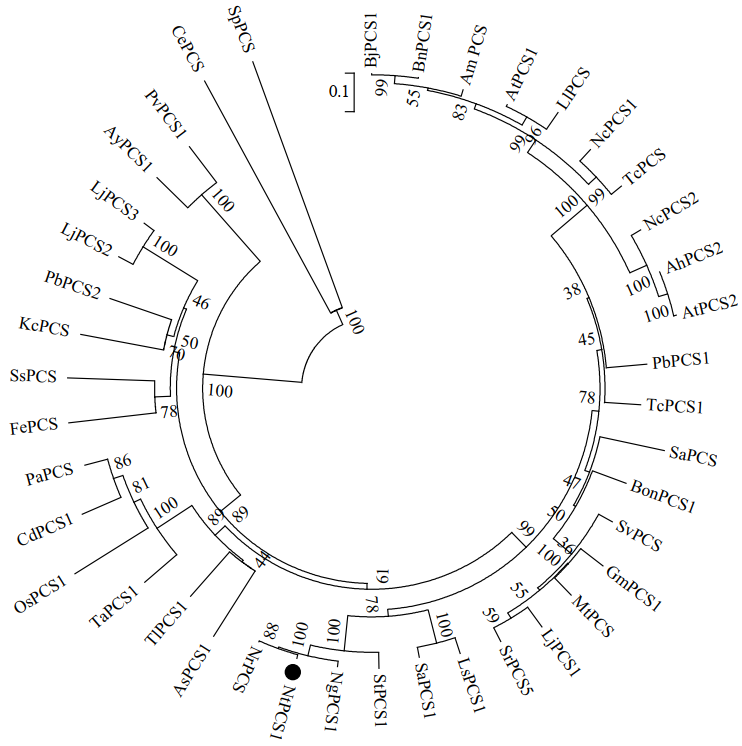


**Supplementary Figure 2.** The phylogentic tree of PCS protein sequences. The neighbor-joining tree (bootstrap method 1000) was drawn with MEGA 6 software. Amino acid sequences of PCS proteins from various species are retrieved from the NCBI database. AhPCS2 (*Arabidopsis halleri*, ADZ24787.1), AmPCS (*Amaranthus tricolor*, AEW23125.1), AsPCS1 (*Allium sativum*, AAO13809.1), AtPCS1 (*Arabidopsis thaliana*, AAD50593.1), AtPCS2 (*Arabidopsis thaliana*, AAK94671.1), AyPCS1 (*Athyrium yokoscense*, BAB64932.1), BjPCS1 (*Brassica juncea*, CAC37692.1), BnPCS1 (*Brassica napus*, CAK24968.2), BonPCS1 (*Boehmeria nivea*, AHC98018.1), CdPCS1 (*Cynodon dactylon*, AAO13810.2), CePCS (*Caenorhabditis elegans*, AAK62992.1), FePCS (*Fagopyrum esculentum*, BAF75863.1), GmPCS1 (*Glycine max*, AAL78384.1), KcPCS (*Kandelia candel*, ADK61091.1), LjPCS1 (*Lotus japonicas*, AAQ01752.1), LjPCS2 (*Lotus japonicas*, AAX99139.1), LjPCS3 (*Lotus japonicas*, AAY81940.1), LlPCS (*Leucaena leucocephala*, ACL00594.3), LsPCS1 (*Lactuca sativa*, AAU93349.1), MtPCS (*Medicago truncatula*, KEH23948.1), NcPCS1 (*Noccaea caerulescens*, AAT07467.1), NcPCS2 (*Noccaea caerulescens*, ABY89660.1), NgPCS1 (*Nicotiana glauca*, ABX10958.1), NrPCS (*Nicotiana rustica*, AJU57239.1), NtPCS1(*Nicotiana tabacum*, AAO74500.1), OsPCS1 (*Oryza sativa*, AAO13349.2), PaPCS (*Phragmites australis*, AFU06381.1), PbPCS1 (*Pyrus betulifolia*, AEY68568.1), PbPCS2 (*Pyrus betulifolia*, AHM93477.1), PvPCS1 (*Pteris vittata*, AAT11885.1), SaPCS (*Sedum alfredii*, AHB86971.1), SaPCS1 (*Sonchus arvensis*, ACU44656.1), SpPCS (*Schizosaccharomyces pombe*, CAA92263.1), SrPCS5 (*Sesbania rostrate*, AAY83876.1), SsPCS (*Suaeda salsa*, AGC82138.1), StPCS1 (*Solanum tuberosum*, CAD68110.1), SvPCS (*Sophora viciifolia*, AFM38979.1), TaPCS1 (*Triticum aestivum*, AAD50592.1), TcPCS (*Thlaspi caerulescens*, BAB93120.1), TcPCS1 (*Theobroma cacao*, XP_007050223.1), TlPCS1 (*Typha latifolia*, AAG22095.3).

**Supplementary Figure 3.** Effect of CdCl_2_ treatment time on transcript level of *NtPCS1* in the WT shoots. 35-day-old WT seedlings were transferred to a fresh 1/2 Hoagland solution containing 0 or 60 μM CdCl_2_, and grown for additional 3, 5 and 7 days. Transcript level of *NtPCS1* was detected using RT-PCR. Values represent means ± SD of three biological replicates. Different letters indicate statistically significant differences (two-way ANOVA followed by a Tukey’s HSD test, *P* < 0.05).


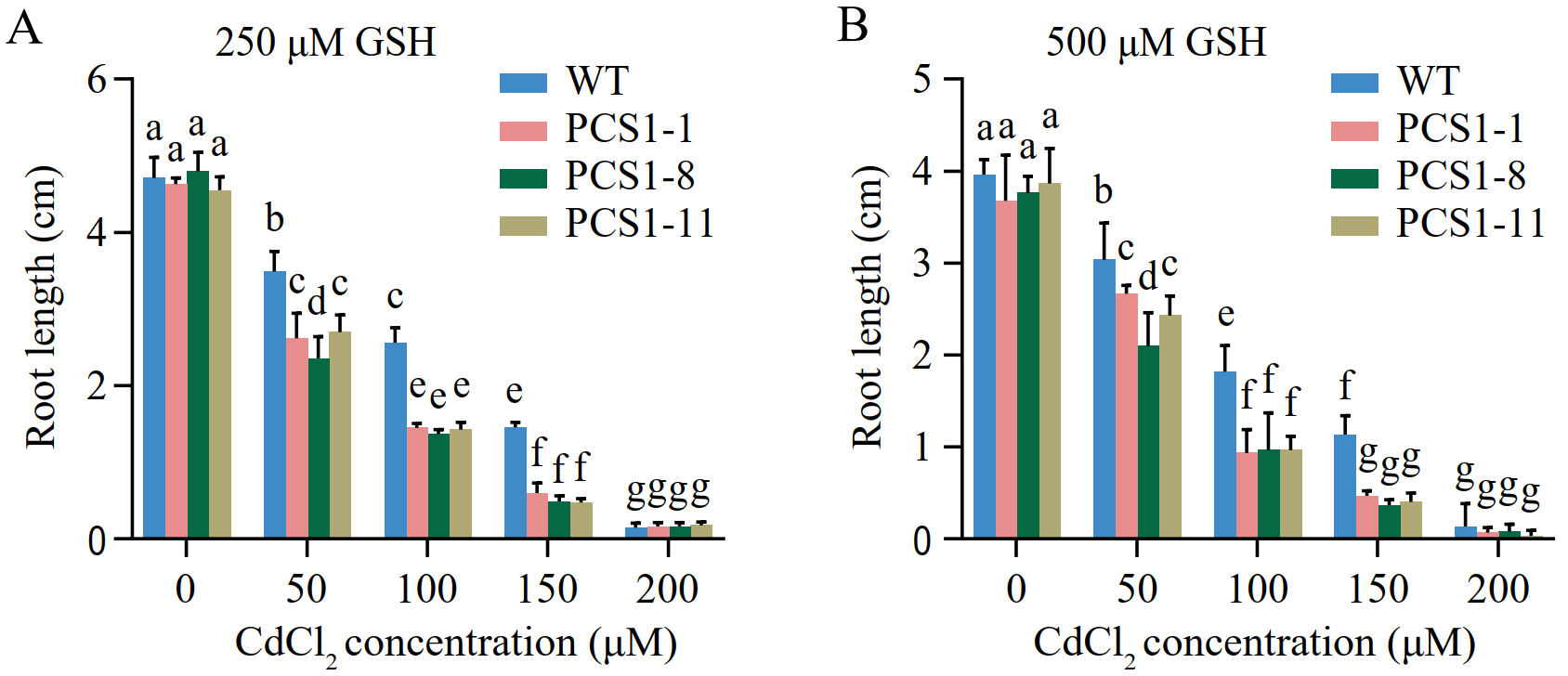


**Supplementary Figure 4.** Effect of CdCl_2_ and GSH on root growth of WT and PCS1 lines. (A, B) Root length of WT and PCS1 lines under various concentrations of CdCl_2_ (0, 50, 100, 150, 200 μM) in the presence of 250 μM (A) or 500 μM (B) GSH. Tobacco seeds were germinated and grown in a vertical orientation on 1/2 MS medium containing CdCl_2_ and GSH. After 21 days, the root length of WT and PCS1 lines was measured. Values represent means ± SD (n = 24). Different letters indicate statistically significant differences (two-way ANOVA followed by a Tukey’s HSD test, *P* < 0.05).


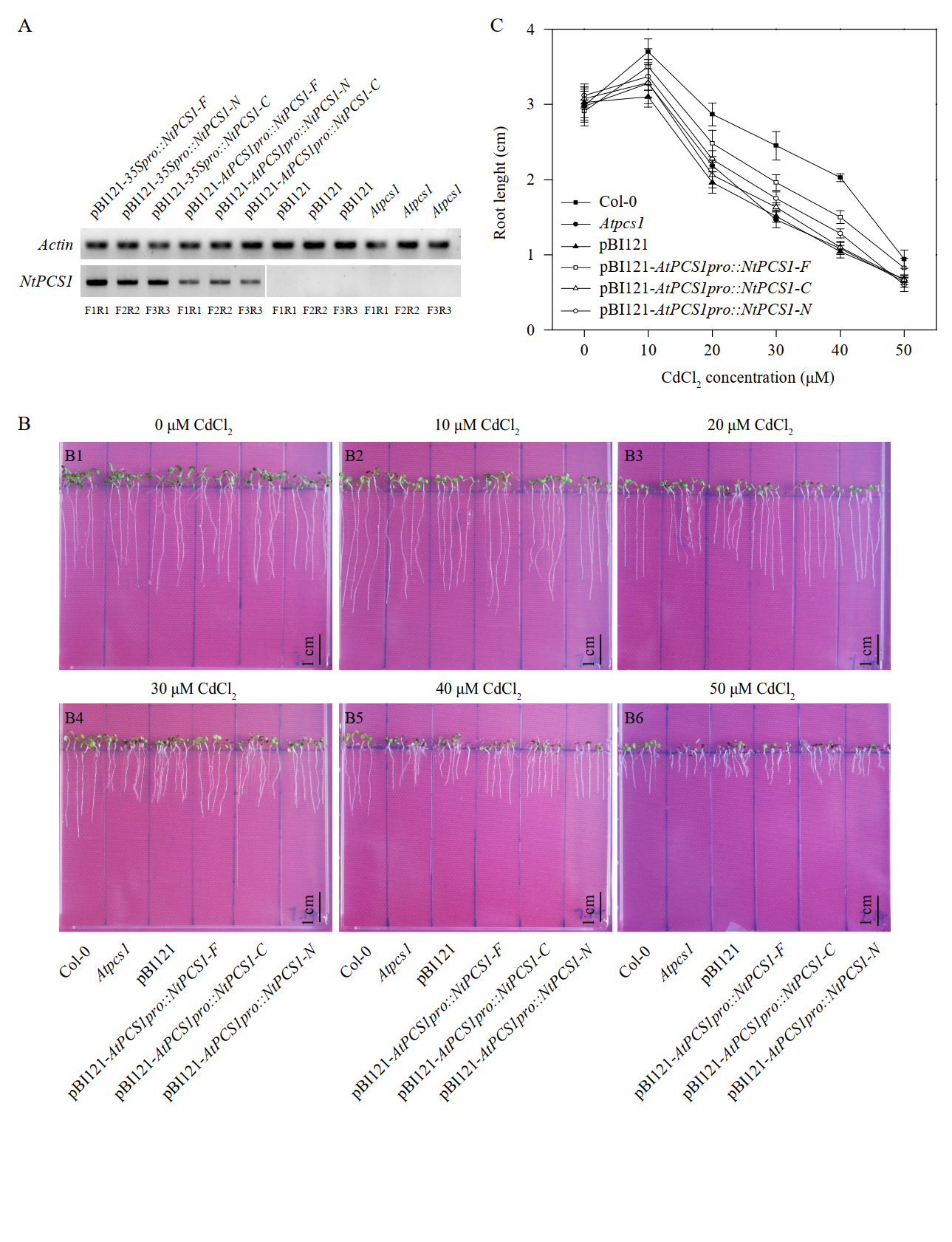


**Supplementary Figure 5.** Functional complementation of the *A. thaliana* *Atpcs1* mutant by heterologous expression of *NtPCS1*. (A) Semi-quantitative RT-PCR was performed on transgenic *A. thaliana* seedlings to detect the transgene. The *Actin* gene was used as a control. Primers F1R1, F2R2, and F3R3 were used to detect the expression levels of the full-length, N-terminal, and C-terminal transgenes of *NtPCS1*, respectively. (B, C) Phenotypes (B) and root length (C) of Col-0, *Atpcs1* mutant and transgenic *A. thaliana* seedlings expressing pBI121-*AtPCS1pro::NtPCS1-F/N/C* treated with 0, 10, 20, 30, 40 or 50 μM CdCl_2_. The *A. thaliana* seeds were germinated and grown in a vertical orientation on 1/2 MS medium containing CdCl_2_. Root length was measured after 9 days. NtPCS1-F represents the full-length of NtPCS1 (1-501 aa), NtPCS1-N represents the N-terminal region of NtPCS1(1-219 aa), and NtPCS1-C represents the C-terminal region of NtPCS1 (220-501 aa). Col-0: Columbia-0; *Atpcs1*: *Atpcs1* mutant; pBI121: *Atpcs1* mutant transformed with empty pBI121 vector; pBI121-*AtPCS1pro::NtPCS1-F/N/C*: *Atpcs1* mutant transformed with pBI121-*AtPCS1pro::NtPCS1-F/N/C*, respectively. Values correspond to means ± SD (n = 20).

**Supplementary Table 1.** Sequence identity of PCS protein sequences determined by BioEdit software. Amino acid sequences of PCS proteins from various species are retrieved from the NCBI database.

**Supplementary Table 2.** Full list of putative regulatory DNA elements of the 1,097 bp promoter region of *NtPCS1* and *AtPCS1* genes. Light green and blue shading represent specific regulatory elements in the promoter region of *NtPCS*1 and *AtPCS1*, respectively.

| ***NtPCS1* promoter** | | | | |
| --- | --- | --- | --- | --- |
| **Name** | **Position** | **Strand** | **Sequence** | **Function** |
| ACE | 409 | + | CTAACGTATT | cis-acting element involved in light responsiveness |
| ACE | 699 | - | AAAACGTTTA | cis-acting element involved in light responsiveness |
| AE-box | 505 | + | AGAAACTT | part of a module for light response |
| ATCT-motif | 291 | - | AATCTAATCC | part of a conserved DNA module involved in light responsiveness |
| ATCT-motif | 656 | + | AATCTGATCG | part of a conserved DNA module involved in light responsiveness |
| Box 4 | 335 | + | ATTAAT | part of a conserved DNA module involved in light responsiveness |
| Box 4 | 617 | - | ATTAAT | part of a conserved DNA module involved in light responsiveness |
| Box I | 515 | - | TTTCAAA | light responsive element |
| CAAT-box | 30 | + | CAAT | common cis-acting element in promoter and enhancer regions |
| CAAT-box | 66 | - | CAAAT | common cis-acting element in promoter and enhancer regions |
| CAAT-box | 78 | - | CAAT | common cis-acting element in promoter and enhancer regions |
| CAAT-box | 140 | - | CAAT | common cis-acting element in promoter and enhancer regions |
| CAAT-box | 176 | - | CAAAT | common cis-acting element in promoter and enhancer regions |
| CAAT-box | 386 | + | CAAAT | common cis-acting element in promoter and enhancer regions |
| CAAT-box | 395 | + | CAAT | common cis-acting element in promoter and enhancer regions |
| CAAT-box | 399 | - | CCAAT | common cis-acting element in promoter and enhancer regions |
| CAAT-box | 441 | - | CAAAT | common cis-acting element in promoter and enhancer regions |
| CAAT-box | 493 | - | CAAT | common cis-acting element in promoter and enhancer regions |
| CAAT-box | 514 | - | CAAAT | common cis-acting element in promoter and enhancer regions |
| CAAT-box | 529 | - | CAAAT | common cis-acting element in promoter and enhancer regions |
| CAAT-box | 581 | + | CAATT | common cis-acting element in promoter and enhancer regions |
| CAAT-box | 607 | + | CAAT | common cis-acting element in promoter and enhancer regions |
| CAAT-box | 710 | - | CAAT | common cis-acting element in promoter and enhancer regions |
| CAAT-box | 714 | + | CAAT | common cis-acting element in promoter and enhancer regions |
| CAAT-box | 809 | - | CAAT | common cis-acting element in promoter and enhancer regions |
| CAAT-box | 872 | - | CAAT | common cis-acting element in promoter and enhancer regions |
| CAAT-box | 897 | - | CAAAT | common cis-acting element in promoter and enhancer regions |
| CAAT-box | 905 | - | CAAT | common cis-acting element in promoter and enhancer regions |
| CAAT-box | 916 | + | CAAT | common cis-acting element in promoter and enhancer regions |
| CAAT-box | 920 | - | CCAAT | common cis-acting element in promoter and enhancer regions |
| CAAT-box | 927 | + | CAAAT | common cis-acting element in promoter and enhancer regions |
| CAAT-box | 1070 | - | CCAAT | common cis-acting element in promoter and enhancer regions |
| GATA-motif | 831 | - | AAGGATAAGG | part of a light responsive element |
| GCN4_motif | 480 | - | TGAGTCA | cis-regulatory element involved in endosperm expression |
| I-box | 765 | - | TATTATCTAGA | part of a light responsive element |
| MBS | 60 | + | CAACTG | MYB binding site involved in drought-inducibility |
| MBS | 162 | + | CAACTG | MYB binding site involved in drought-inducibility |
| Skn-1_motif | 468 | + | GTCAT | cis-acting regulatory element required for endosperm expression |
| Skn-1_motif | 479 | - | GTCAT | cis-acting regulatory element required for endosperm expression |
| Sp1 | 255 | - | CC(G/A)CCC | light responsive element |
| TATA-box | 73 | + | TATA | core promoter element around -30 of transcription start |
| TATA-box | 144 | + | TTTTA | core promoter element around -30 of transcription start |
| TATA-box | 361 | - | TATAA | core promoter element around -30 of transcription start |
| TATA-box | 362 | + | TATA | core promoter element around -30 of transcription start |
| TATA-box | 377 | - | TAATA | core promoter element around -30 of transcription start |
| TATA-box | 429 | + | TTTTA | core promoter element around -30 of transcription start |
| TATA-box | 500 | + | ATTATA | core promoter element around -30 of transcription start |
| TATA-box | 501 | - | TATAA | core promoter element around -30 of transcription start |
| TATA-box | 502 | + | TATA | core promoter element around -30 of transcription start |
| TATA-box | 564 | - | TATA | core promoter element around -30 of transcription start |
| TATA-box | 566 | + | TAATA | core promoter element around -30 of transcription start |
| TATA-box | 574 | + | TTTTA | core promoter element around -30 of transcription start |
| TATA-box | 613 | - | TATAA | core promoter element around -30 of transcription start |
| TATA-box | 614 | - | TATA | core promoter element around -30 of transcription start |
| TATA-box | 616 | - | TAATA | core promoter element around -30 of transcription start |
| TATA-box | 624 | + | TTTTA | core promoter element around -30 of transcription start |
| TATA-box | 653 | - | TTTTA | core promoter element around -30 of transcription start |
| TATA-box | 680 | - | TATA | core promoter element around -30 of transcription start |
| TATA-box | 706 | + | TTTTA | core promoter element around -30 of transcription start |
| TATA-box | 738 | - | TTTTA | core promoter element around -30 of transcription start |
| TATA-box | 771 | - | TTTTA | core promoter element around -30 of transcription start |
| TATA-box | 833 | - | TATAA | core promoter element around -30 of transcription start |
| TATA-box | 834 | - | TATA | core promoter element around -30 of transcription start |
| TATA-box | 845 | - | TAATA | core promoter element around -30 of transcription start |
| TATA-box | 862 | - | ATATAT | core promoter element around -30 of transcription start |
| TATA-box | 863 | - | TATA | core promoter element around -30 of transcription start |
| TATA-box | 977 | + | TTTTA | core promoter element around -30 of transcription start |
| TATA-box | 999 | + | TAATA | core promoter element around -30 of transcription start |
| TATA-box | 1016 | - | TTTTA | core promoter element around -30 of transcription start |
| TC-rich repeats | 303 | - | GTTTTCTTAC | cis-acting element involved in defense and stress responsiveness |
| WUN-motif | 348 | + | TCATTACGAA | wound-responsive element |
| ***AtPCS1* promoter** | | | | |
| **Name** | **Position** | **Strand** | **Sequence** | **Function** |
| AAAC-motif | 706 | + | CAACAAAAACCT | light responsive element |
| ARE | 541 | - | TGGTTT | cis-acting regulatory element essential for the anaerobic induction |
| ARE | 712 | - | TGGTTT | cis-acting regulatory element essential for the anaerobic induction |
| ARE | 655 | - | TGGTTT | cis-acting regulatory element essential for the anaerobic induction |
| ATC-motif | 392 | - | AGCTATCCA | part of a conserved DNA module involved in light responsiveness |
| ATGCAAAT motif | 22 | - | ATACAAAT | cis-acting regulatory element associated to the TGAGTCA motif |
| Box 4 | 788 | - | ATTAAT | part of a conserved DNA module involved in light responsiveness |
| Box I | 132 | + | TTTCAAA | light responsive element |
| Box-W1 | 598 | + | TTGACC | fungal elicitor responsive element |
| Box-W1 | 893 | + | TTGACC | fungal elicitor responsive element |
| Box-W1 | 863 | - | TTGACC | fungal elicitor responsive element |
| Box-W1 | 910 | + | TTGACC | fungal elicitor responsive element |
| CAAT-box | 8 | + | CAATT | common cis-acting element in promoter and enhancer regions |
| CAAT-box | 22 | - | CAAAT | common cis-acting element in promoter and enhancer regions |
| CAAT-box | 42 | + | CAAAT | common cis-acting element in promoter and enhancer regions |
| CAAT-box | 72 | - | CAAT | common cis-acting element in promoter and enhancer regions |
| CAAT-box | 88 | + | CAAT | common cis-acting element in promoter and enhancer regions |
| CAAT-box | 135 | + | CAAAT | common cis-acting element in promoter and enhancer regions |
| CAAT-box | 211 | + | CAAT | common cis-acting element in promoter and enhancer regions |
| CAAT-box | 308 | - | CAAT | common cis-acting element in promoter and enhancer regions |
| CAAT-box | 312 | + | CAAT | common cis-acting element in promoter and enhancer regions |
| CAAT-box | 330 | + | CAAAT | common cis-acting element in promoter and enhancer regions |
| CAAT-box | 341 | + | CAATT | common cis-acting element in promoter and enhancer regions |
| CAAT-box | 343 | - | CAAAT | common cis-acting element in promoter and enhancer regions |
| CAAT-box | 349 | - | CAAT | common cis-acting element in promoter and enhancer regions |
| CAAT-box | 357 | - | CAAT | common cis-acting element in promoter and enhancer regions |
| CAAT-box | 362 | + | CAATT | common cis-acting element in promoter and enhancer regions |
| CAAT-box | 440 | + | CCAAT | common cis-acting element in promoter and enhancer regions |
| CAAT-box | 441 | + | CAAT | common cis-acting element in promoter and enhancer regions |
| CAAT-box | 472 | - | CAAT | common cis-acting element in promoter and enhancer regions |
| CAAT-box | 478 | - | CAAAT | common cis-acting element in promoter and enhancer regions |
| CAAT-box | 488 | + | CAAAT | common cis-acting element in promoter and enhancer regions |
| CAAT-box | 506 | - | CAATT | common cis-acting element in promoter and enhancer regions |
| CAAT-box | 507 | - | CAAT | common cis-acting element in promoter and enhancer regions |
| CAAT-box | 514 | - | CAAT | common cis-acting element in promoter and enhancer regions |
| CAAT-box | 518 | - | CAAT | common cis-acting element in promoter and enhancer regions |
| CAAT-box | 536 | + | CAAAT | common cis-acting element in promoter and enhancer regions |
| CAAT-box | 630 | + | CAAT | common cis-acting element in promoter and enhancer regions |
| CAAT-box | 716 | + | CAAAT | common cis-acting element in promoter and enhancer regions |
| CAAT-box | 723 | + | CAAT | common cis-acting element in promoter and enhancer regions |
| CAAT-box | 782 | - | CAATT | common cis-acting element in promoter and enhancer regions |
| CAAT-box | 783 | - | CAAT | common cis-acting element in promoter and enhancer regions |
| CAAT-box | 909 | - | CAAT | common cis-acting element in promoter and enhancer regions |
| CAAT-box | 926 | + | CAAAT | common cis-acting element in promoter and enhancer regions |
| CAAT-box | 931 | - | CAATT | common cis-acting element in promoter and enhancer regions |
| CAAT-box | 932 | - | CAAT | common cis-acting element in promoter and enhancer regions |
| CAAT-box | 959 | + | CAAAT | common cis-acting element in promoter and enhancer regions |
| CAAT-box | 973 | + | CCCAATTT | common cis-acting element in promoter and enhancer regions |
| CAAT-box | 974 | + | CCAAT | common cis-acting element in promoter and enhancer regions |
| CAAT-box | 975 | + | CAATT | common cis-acting element in promoter and enhancer regions |
| CGTCA-motif | 627 | + | CGTCA | cis-acting regulatory element involved in the MeJA-responsiveness |
| CGTCA-motif | 857 | - | CGTCA | cis-acting regulatory element involved in the MeJA-responsiveness |
| G-box | 618 | - | GACATGTGGT | cis-acting regulatory element involved in light responsiveness |
| G-box | 622 | + | CACGTC | cis-acting regulatory element involved in light responsiveness |
| GAG-motif | 918 | + | AGAGAGT | part of a light responsive element |
| GT1-motif | 680 | - | GGTTAA | light responsive element |
| LAMP-element | 652 | + | CCAAAACCA | part of a light responsive element |
| LTR | 431 | + | CCGAAA | cis-acting element involved in low-temperature responsiveness |
| LTR | 994 | + | CCGAAA | cis-acting element involved in low-temperature responsiveness |
| LTR | 729 | + | CCGAAA | cis-acting element involved in low-temperature responsiveness |
| MBSII | 111 | - | AAAAGTTAGTTA | MYB binding site involved in flavonoid biosynthetic genes regulation |
| Skn-1_motif | 92 | + | GTCAT | cis-acting regulatory element required for endosperm expression |
| Skn-1_motif | 640 | + | GTCAT | cis-acting regulatory element required for endosperm expression |
| Skn-1_motif | 221 | + | GTCAT | cis-acting regulatory element required for endosperm expression |
| TATA-box | 36 | + | TAATA | core promoter element around -30 of transcription start |
| TATA-box | 51 | - | TTTTA | core promoter element around -30 of transcription start |
| TATA-box | 77 | + | ATATAT | core promoter element around -30 of transcription start |
| TATA-box | 78 | + | TATA | core promoter element around -30 of transcription start |
| TATA-box | 80 | + | TATA | core promoter element around -30 of transcription start |
| TATA-box | 109 | - | TTTTA | core promoter element around -30 of transcription start |
| TATA-box | 151 | - | TTTTA | core promoter element around -30 of transcription start |
| TATA-box | 157 | - | TATAA | core promoter element around -30 of transcription start |
| TATA-box | 158 | + | TATA | core promoter element around -30 of transcription start |
| TATA-box | 169 | - | TAATA | core promoter element around -30 of transcription start |
| TATA-box | 179 | + | TATA | core promoter element around -30 of transcription start |
| TATA-box | 288 | + | TAATA | core promoter element around -30 of transcription start |
| TATA-box | 353 | + | TAATA | core promoter element around -30 of transcription start |
| TATA-box | 371 | - | TTTTA | core promoter element around -30 of transcription start |
| TATA-box | 375 | - | TATATGT | core promoter element around -30 of transcription start |
| TATA-box | 377 | + | ATATAAT | core promoter element around -30 of transcription start |
| TATA-box | 378 | + | TATA | core promoter element around -30 of transcription start |
| TATA-box | 421 | - | TTTTA | core promoter element around -30 of transcription start |
| TATA-box | 696 | - | TTTTA | core promoter element around -30 of transcription start |
| TATA-box | 736 | - | TTTTA | core promoter element around -30 of transcription start |
| TATA-box | 750 | - | TTTTA | core promoter element around -30 of transcription start |
| TATA-box | 760 | + | TAATA | core promoter element around -30 of transcription start |
| TATA-box | 787 | - | TAATA | core promoter element around -30 of transcription start |
| TATA-box | 817 | + | TAATA | core promoter element around -30 of transcription start |
| TATA-box | 942 | + | tcTATATAtt | core promoter element around -30 of transcription start |
| TATA-box | 944 | - | TATACA | core promoter element around -30 of transcription start |
| TATA-box | 946 | - | TATA | core promoter element around -30 of transcription start |
| TATA-box | 948 | - | TAATA | core promoter element around -30 of transcription start |
| TATA-box | 1067 | - | TATATATA | core promoter element around -30 of transcription start |
| TATA-box | 1068 | - | ATATAT | core promoter element around -30 of transcription start |
| TATA-box | 1069 | - | TATA | core promoter element around -30 of transcription start |
| TATA-box | 1071 | - | TATA | core promoter element around -30 of transcription start |
| TGACG-motif | 627 | - | TGACG | cis-acting regulatory element involved in the MeJA-responsiveness |
| TGACG-motif | 857 | + | TGACG | cis-acting regulatory element involved in the MeJA-responsiveness |
| W box | 598 | + | TTGACC |  |
| W box | 893 | + | TTGACC |  |
| W box | 863 | - | TTGACC |  |
| W box | 910 | + | TTGACC |  |
| WUN-motif | 756 | - | TCATTACGAA | wound-responsive element |
| circadian | 849 | - | CAANNNNATC | cis-acting regulatory element involved in circadian control |
